# Significant Tumor Inhibition of Trimethyl‐15^2^‐[L‐aspartyl]pheophorbide a in Tumor Photodynamic Therapy^≠^

**DOI:** 10.1002/cmdc.202500087

**Published:** 2025-04-11

**Authors:** Anita Benić, Akmaral Kussayeva, Ivana Antol, Mario Vazdar, Zlatko Brkljača, Dan‐Ye Chen, Yi‐Jia Yan, Ying‐Hua Gao, Zhi‐Long Chen, Davor Margetić

**Affiliations:** ^1^ Division of Organic Chemistry and Biochemistry Ruđer Bošković Institute Bijenička c. 54 Zagreb 10000 Croatia; ^2^ Department of Mathematics Informatics and Cybernetics University of Chemistry and Technology 16628 Prague Czech Republic; ^3^ Department of Pharmaceutical Science & Technology Donghua University Shanghai 201620 China; ^4^ Department of Orthopedics Shanghai Bone Tumor Institution Shanghai General Hospital Shanghai Jiao Tong University School of Medicine 100 Haining Street Shanghai 200080 China; ^5^ Present address: Selvita d.o.o. Prilaz baruna Filipovića 29 10000 Zagreb Croatia

**Keywords:** photodynamic therapy, cancer, pheophorbide, molecular dynamics, keyword 5

## Abstract

A novel pheophorbide derivative, trimethyl‐15^2^‐[L‐aspartyl]pheophorbide a was synthesised and investigated for anti‐tumor activity. The prepared photosensitizer had good absorption in the phototherapeutic window and high ROS yields. It exhibited excellent phototoxicity higher than reference compound *m*‐THPC when irradiated by 650 nm light in vitro, and obvious photodynamic anti‐tumor effect in vivo. It causes cellular apoptosis or necrosis under laser irradiation and localizes in mitochondria, lysosome, and endoplasmic reticulum. The observed high efficacy was rationalized by efficient introduction into the blood by facile transfer through membranes, which is supported by molecular dynamics simulation studies. This work provides a new candidate photosensitizer for anti‐cancer treatment.

## Graphical abstract

Novel pheophorbide A, a chlorophyll‐derived compound was prepared by simple and sustainable synthesis. Small structural change to known molecules has a profound effect on the PDT efficacy and its phototoxicity is substantially higher than reference compound *m*‐THPC. In vitro and in vivo results are supplemented with molecular dynamics studies on its better membrane permeability.

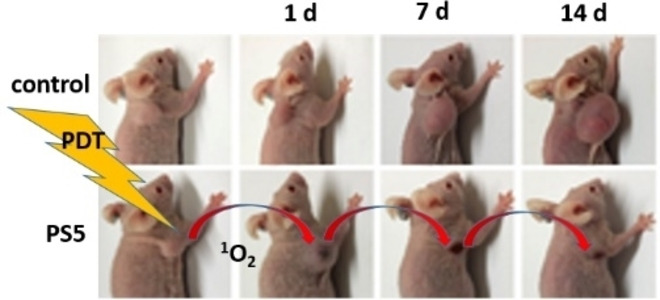

## Introduction

Photodynamic therapy (PDT) has become one of the alternative clinical cancer treatment modalities besides surgery, radiotherapy, chemotherapy, and biological immunotherapy.^1,2^ In PDT, the destruction of various cells and tissues is based on the photochemical conversion of oxygen by the activation with light photosensitizer (PS), leading to the generation of reactive oxygen species (ROS).^3,4^ This treatment has been used in several medical fields, such as antitumor, antimicrobial, and antiviral.^5−8^ In addition, the main advantages of ideal photosensitizers should have a high extinction coefficient in the visible or near‐infrared (NIR) region (600‐850 nm), high selectivity in target tissues, high yields of active oxygen, low dark toxicity, and high photo‐toxicity.^9^ Chlorophyll derivatives have attracted great attention in PDT treatment as photosensitizers since they combine desirable spectral characteristics, low toxicity, and tumor‐collective effects.^10^ Pheophorbide *a*, is a chlorophyll derivative, which showed antitumor effects in several human cancers such as hepatocellular carcinoma^11^ and prostate cancer,^12^ and combination therapy.^13,14^ Their potential in photodynamic therapy led to the synthesis of various derivatives of pheophorbide *a* ^15−17^ and it was shown that even small changes in molecular structure could have an important influence on their properties in PDT. In continuation of our studies of chlorophyll sensitizers for the applications in PDT,^18−22^ here we report the preparation of a novel derivative of pheophorbide *a* and the evaluation of its PDT activity *in vitro* and *in vivo*.

**Figure 1:**
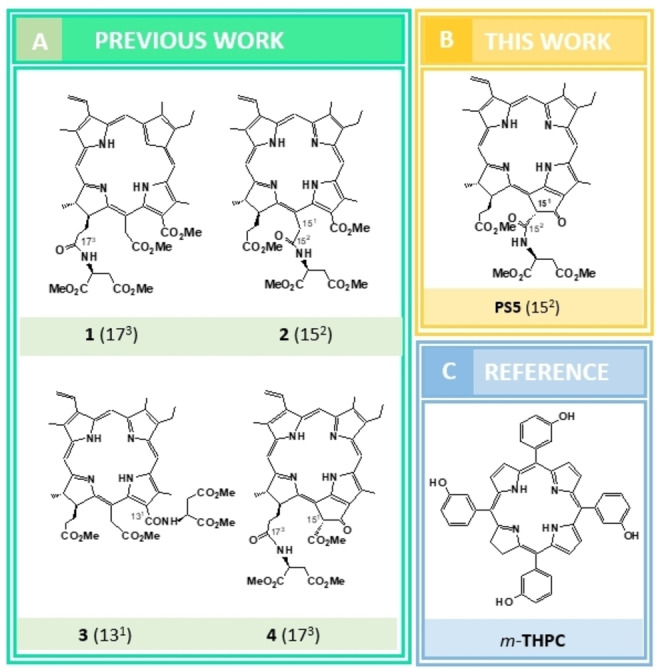
Previously reported monoaspartyl chlorin *e*_6_ and pheophorbide *a* derivatives, novel aspartyl pheophorbide *a* photosensitizer **PS5** and reference molecule.

Synthesis of three monoaspartyl chlorin *e*_6_ derivatives **1–3** (17^3^‐, 15^2^‐, and 13^1^−) as well as 17^3^‐derivative of pheophorbide *a* (trimethyl‐17^3^‐[L‐aspartyl]pheophorbide *a* **(4**) was described by Smith et al.^22^ (Figure 1).

Here we describe the preparation of novel trimethyl‐15^2^‐[L‐aspartyl] pheophorbide a **PS5**, which is an isomeric derivative of pheophorbide **4**, and compare its properties with reference photosensitizer *m*‐tetra(hydroxyphenyl)chlorin (*m*‐THPC, Foscan, Temoporfin).

## Results and Discussion

### Synthesis

**Scheme 1:**
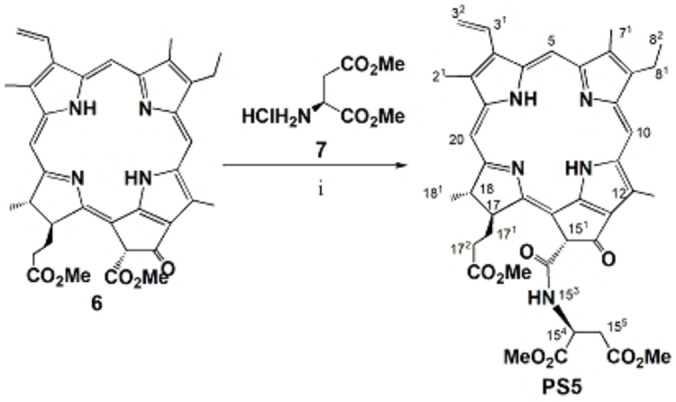
Synthesis of **PS5**. Conditions: i) dry toluene, 110 °C, 2 h, 90 %

The synthesis of novel pheophorbide a derivative was designed because of its simplicity and the advantageous employment of semi‐synthesis from naturally abundant material. In such a way, larger quantities of target molecules could be provided from renewable sources and are viable for the envisaged possibility of up‐scaling. Hence, dimethyl pheophorbide a **6** was isolated from Spirulina pacifica algae in one reaction step by modified literature procedure.^23,24^ Then, trimethyl‐15^2^‐[L‐aspartyl] pheophorbide a **PS5** was prepared in one reaction step in 90 % yield by reaction of dimethyl pheophorbide a **6** with dimethyl L‐aspartate hydrochloride **7** following the thermal procedure of Belykh et al.^25^ which was modified for L‐aspartate (Scheme 1). Obtained compound **PS5** was fully spectroscopically characterized and the structural assignments were based on the 1D and 2D NMR spectroscopy.

### Photophysical Properties

**Figure 2:**
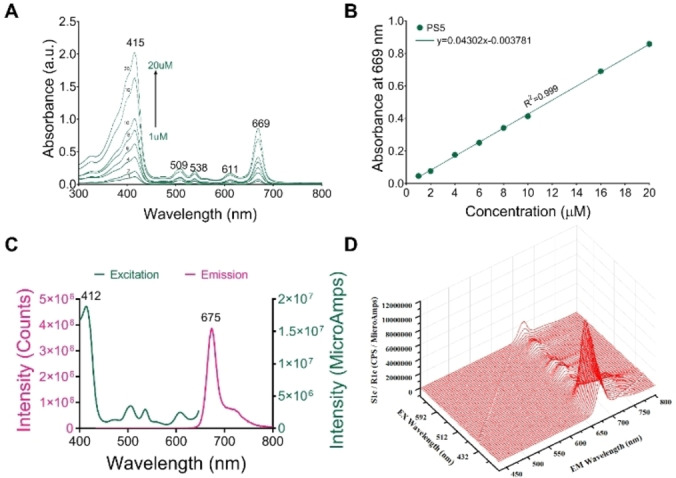
Photophysical properties **of PS5**. A: Absorption spectra of **PS5** with different concentrations (1 μM ~20 μM) in DMSO. B: Linear correlation curve between the drug concentration and absorbance of compound **PS5**. C: The fluorescence excitation spectrum of **PS5** in DMSO and the fluorescence spectrum recorded with an excitation at 412 nm. D: The 3D fluorescence matrix of **PS5**.

The optimal therapeutic window of an ideal photosensitizer is 600–850 nm for PDT.^26^ Therefore, research and development of photosensitizers with longer absorption wavelengths in the near‐infrared region is an important goal in photodynamic therapy. The electronic absorption spectrum of compound **PS5** was recorded in DMSO. The result showed that **PS5** conformed to the absorption peak characteristic of the chlorin e_6_‐based photosensitizers. Compound **PS5** displays a strong absorption of the Soret band at 415 nm (ϵ~10^5^ L/mol^−1^ cm^−1^) and several Q bands from 500 to 700 nm (Figure 2A, Table 1). In addition, compound **PS5** has a positive correlation between drug absorption and concentration in 1 μM ~20 μM (Figure 2B). Figure 2C displays the fluorescence spectra of **PS5** in DMSO whose emission wavelength was 675 nm. The 3D fluorescence matrix intuitively showed the fluorescence intensity of the compound **PS5** (Figure 2D).

**Table 1:** Summary of the photophysical and photochemical data of 10 μM of **PS5** in DMSO solution.

λ_max_ (nm) (ϵ)	λ_ex_ (nm)	λ_em_ (nm)	Φ_Δ_^a^
1415(100700); 509(10400); 538(8400); 611(6900); 669(41300)	412	674	1.19

[a] Using Rose Bengal in DMF as the reference (Φ_Δ_=0.47 in DMF).

#### Photobleaching

**Figure 3:**
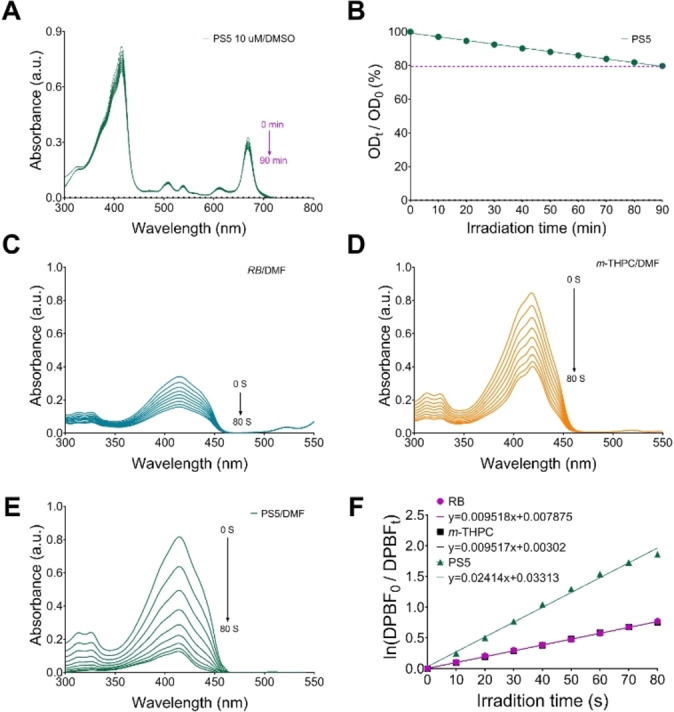
Photophysical properties of **PS5**. A: The changes in absorbance under 650 nm irradiation. B: The bleaching rate of **PS5** under 650 nm irradiation. C: Photodecomposition of DPBF by ^1^O_2_ after irradiation of RB in DMF (RB=Rose Bengal). D: Photodecomposition of DPBF by ^1^O_2_ after irradiation of *m*‐THPC in DMF. E: Photodecomposition of DPBF by ^1^O_2_ after irradiation of **PS5** in DMF. F: The plot for the generation rate of ^1^O_2_ (**PS5**, RB, *m*‐THPC) in DMF.

Photobleaching is one of the important factors affecting the photodynamic effect of photosensitizers.^27^ The absorbance of the compound did not decrease significantly after irradiation (Figures 3A and 3B), and this result indicated that the structure of compound **PS5** was stable and did not easily undergo photodegradation.

#### Singlet Oxygen Generation

The ability of singlet oxygen generation of the new pheophorbide derivative was determined by 1,3‐diphenylisobenzofuran (DPBF) as a singlet oxygen capture agent. The changes in absorbance intensity at 417 nm in DMF solution determined the state of singlet oxygen capacity. The photo‐oxidation effect was obvious under short‐time illumination. The compound **PS5** had a relatively fast decrease in absorbance compared to *m*‐THPC (Figures 3C‐3E). It is known that at 650 nm laser irradiation, the generation rate of singlet oxygen in *m*‐THPC is comparable to Rose Bengal (Φ_Δ_=0.47).^28^ The singlet oxygen quantum yield of **PS5** (Φ_Δ_=1.19, Table 1) was 2.5‐fold compared to *m‐*THPC (Figure 3F). The high ^1^O_2_ quantum yield of **PS5** shows its capacity to be applied as an effective phototherapeutic agent.

#### Cytotoxicity

**Figure 4:**
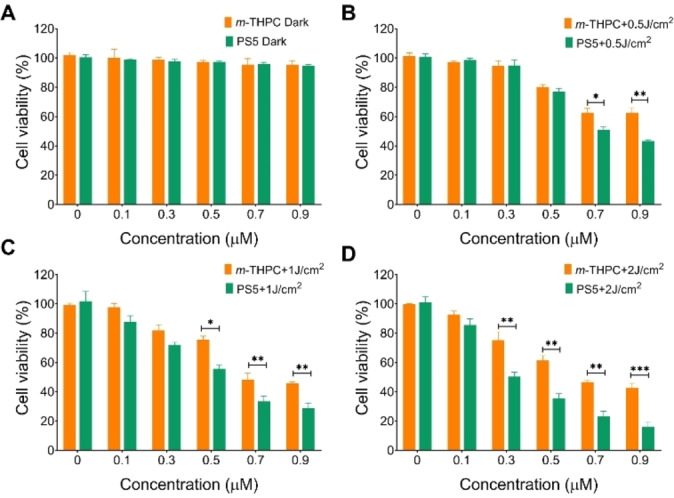
Dark toxicity and photo‐toxicity of photosensitizers (**PS5**, and *m*‐THPC). A: The dark toxicity of photosensitizers **PS5** and *m‐*THPC. B: The cell viability of **PS5** and *m*‐THPC under 0.5 J/cm^2^ irradiation. C: The cell viability of photosensitizers **PS5** and *m*‐THPC under 1 J/cm^2^ irradiation. D: The cell viability of photosensitizer **PS5** and *m*‐THPC under 1.5 J/cm^2^ irradiation *P <0.05, **P<0.01, ***P <0.001,compared to controls.

The dark cytotoxicity and photo‐toxicity of compound **PS5** and the reference compound *m*‐THPC were evaluated in A549 cells. The results are shown in Figure 4 and are summarized in Table 2. In the dark, compound **PS5** and *m*‐THPC were found to be nontoxic at lower concentrations (<1 μM) (Figure 4A). However, upon exposure to a low light dose (0.5 J/cm^2^), compound **PS5** was found to be highly toxic to A549 cells, showing a higher anti‐tumor effect than *m*‐THPC in vitro. At low doses of light intensity, the **PS5** and *m*‐THPC showed IC_50_ values of 0.859 and 1.179 μM, respectively. For increased light dose, the IC_50_ values decreased. When the light dose was 1.5 J/cm^2^, the IC_50_ value of *m*‐THPC was about two‐fold greater than **PS5**. These results indicated that photosensitizer **PS5** could produce toxic species to kill tumor cells under irradiation more efficiently than *m*‐THPC.

**Table 2:** Cytotoxicity (A549 cells) of compound **PS5** and *m*‐THPC.

	Phototoxicity (IC^50^, μM)		
Compound	0.5 J/cm^2^	1 J/cm^2^	1.5 J/cm^2^
PS5	0.859	0.505	0.321
*m*‐THPC	1.179	0.816	0.745

#### Cellular Uptake

**Figure 5:**
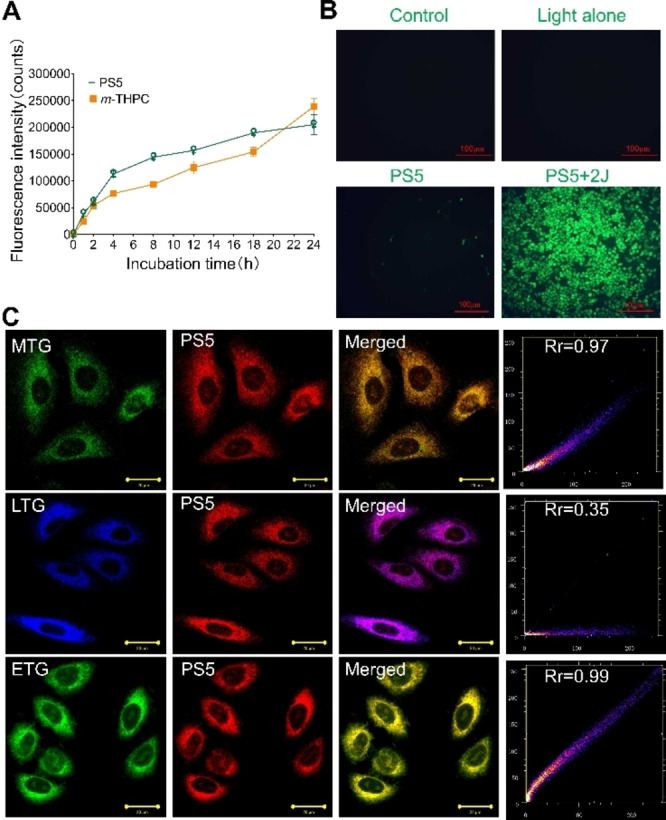
A: Intracellular accumulation of compounds **PS5** and *m*‐THPC in A549 cells. B: ROS fluorescence staining of A549 cells subjected to different treatments. Scale bar: 100 μm. C: The subcellular localization of **PS5** in A549 cells co‐stained with Mito‐Tracker Green (MTG), Lyso‐Tracker Green (LTG), and ER‐Tracker Green (ETG) imaged by confocal laser scanning microscope. Scale bar: 20 μm.

Targeted PDT offers enhanced intracellular accumulation of the photosensitizer, leading to improved PDT efficacy and reduced toxicity to normal tissues.^29^ The intracellular accumulation experiment demonstrated that compound **PS5** exhibits a higher accumulation rate compared to *m*‐THPC, with both compounds accumulating more within cells over time (Figure 5A). A study by Hilf et al.^30^ on the cellular uptake of PDT drug during 24 h showed that the highest accumulation is related to the lowest in vitro phototoxicity. Furthermore, Fedoročko et al.^31^ showed that in addition to the total intracellular level of PS, the ability of the cell to manage the PS‐induced ROS level are crucial factors affecting the final cell response to PDT. It is also suggested that differences in relative subcellular distribution might slightly affect the PDT efficacy.

#### ROS Generation

The mechanism mediated by reactive oxygen species (ROS) is the main reason for the efficacy of PDT. The DCFH‐DA staining results showed the presence of green fluorescence in PS5+2 J group, indicating the generation of ROS in the PDT group. However, in the light alone and **PS5** alone groups the ROS generation was not observed (Figure 5B).

#### Intracellular Localization

The subcellular localization of photosensitizer **PS5** evaluated by a confocal laser scanning microscope has shown that the red fluorescence of the **PS5** could overlap with the probe (Figure 5C). The photosensitizer **PS5** was found to localize in the mitochondria, lysosomes and endoplasmic reticulum and this is consistent with the localization of *m*‐THPC^32^ and photosensitizers reported in the literature.^33,34^

#### Caspase Activity Assay

**Figure 6:**
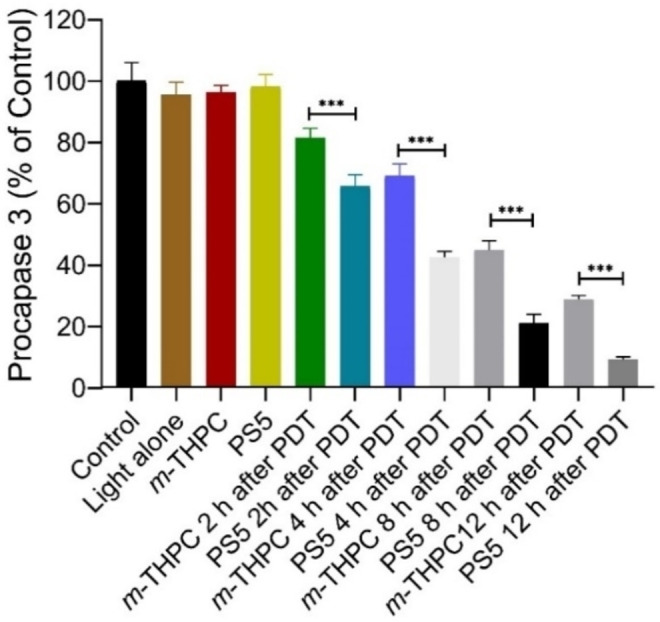
Activation of procaspase‐3 by **PS5** and *m*‐THPC in the cultured A549 cells after PDT, then incubated for different time points (2 h, 4 h, 8 h, and 12 h). The density ratios of the activation of procaspase‐3 were first calculated. The changes were expressed as the percentage of controls (untreated). Data represent mean ± S.D. (n=3), *p <0.05, **p <0.01, ***p <0.001 compared to control.

The activity of caspase‐3 was determined, which had been shown to play a pivotal role in the execution phase of apoptosis induced in PDT.^35^ Under exposure to compounds **PS5** and *m*‐THPC, caspase‐3 activity significantly increased by the incubation 2 h after PDT compared with the control (Figure 6). This finding indicates that PDT induced significant procaspase‐3 degradation and apoptosis of the cultured A549 cells.^36^

#### Therapeutic Effects of the Photosensitizers in vivo

**Figure 7:**
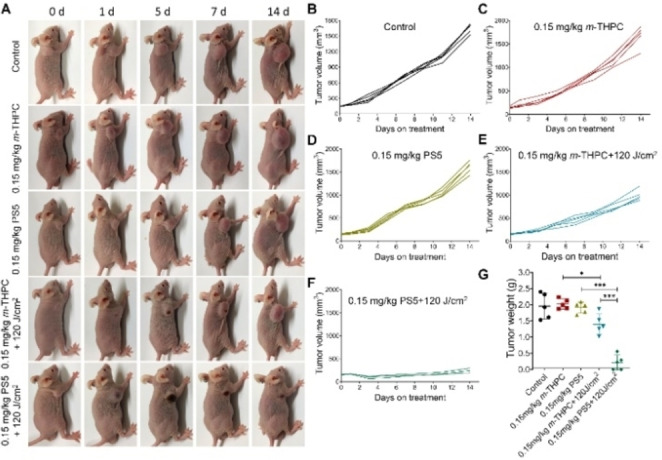
The photodynamic anti‐tumor effect of **PS5** in A549 xenograft tumors. A: PDT efficacy of 0.15 mg/kg of compounds **PS5** and *m*‐THPC in A549 tumor‐bearing Balb/c nude mice. B−F: The tumor volume of mice at different time points. G: The tumor weight for each group at the end of the observation period. Data are presented as the mean ± SD. *p <0.05, **p <0.01, ***p <0.001 compared to control.

To evaluate the in vivo therapeutic efficacy, A549 tumor‐bearing mice were randomly divided into five groups: control, 0.15 mg/kg *m*‐THPC (without laser), 0.15 mg/kg **PS5** (without laser), 0.15 mg/kg *m*‐THPC+120 J/cm^2^ (*m*‐THPC‐PDT) and 0.15 mg/kg **PS5**+120 J/cm^2^ (**PS5**‐PDT). Mice were once intravenously injected with 0.15 mg/kg photosensitizer. After treatment, tumor volumes of each group were recorded every other day (Figures 7B‐7F). Compared to the control group, **PS5**‐PDT showed a significant inhibition effect on tumor growth. After 14 days, the tumors were excised and weighed. The average weight of tumors in the control group was 1.957 g. Importantly, the average weight of tumors in the **PS5**‐PDT group was very low (0.215 g) and there was no change in the drug‐alone group without laser compared to the control group (Figure 7G). As shown in Figure 7A, the higher anti‐tumor effect of compound **PS5** is obvious compared to *m*‐THPC.

**Figure 8:**
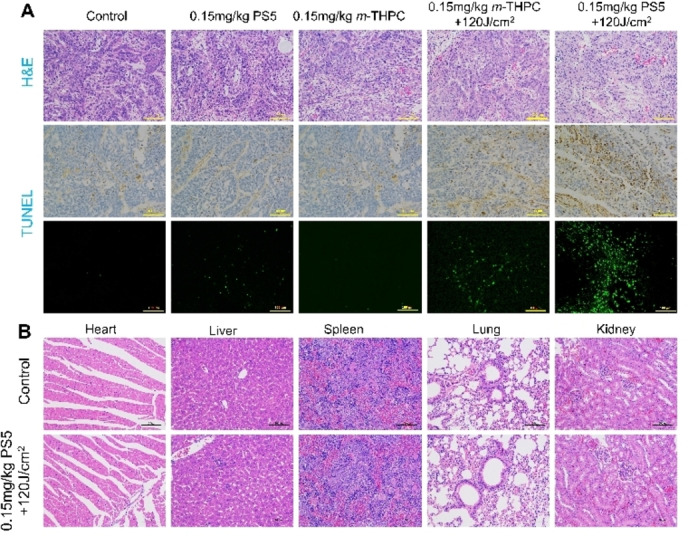
A: Hematoxylin and eosin (H&E) and TUNEL staining images of different treatment groups (control, **PS5**, *m*‐THPC, **PS5**‐PDT, *m*‐THPC‐PDT) of mice. B: H&E staining images of the main organs from groups (control, **PS5**‐PDT) of mice. Scale bar: 100 μm.

Additional information on the effects of **PS5**‐PDT was obtained by the H&E staining and TUNEL fluorescence assay. The results revealed that the light alone group and 0.15 mg/kg **PS5** alone group resulted in few apoptotic cells, whereas the positive apoptosis exhibiting green fluorescence was significantly increased in the 0.15 mg/kg **PS5** + 120 J/cm^2^ group (Figure 8A). Therefore, the **PS5** photosensitizer could be considered to be safe in PDT. These results showed that photosensitizer **PS5** had a better anti‐tumor effect in vivo and holds great promise to be further developed for a clinical study.

The potential in vivo toxicity has always been a problem in the development of photosensitizers which we evaluated in this work. A further indication of the low in vivo toxicity of **PS5** was obtained by analyzing the main organs (heart, liver, spleen, lungs and kidneys) and tumor, which were harvested from mice after 14 days of treatment. The optical microscopy shows that there were no obvious cell damage and morphology changes in normal tissues for the **PS5**‐PDT group compared to the control group (Figure 8B).^37^ However, there was significant necrosis of tumor tissue in the **PS5**‐PDT group.

The high efficacy of the **PS5** derivative can be primarily rationalized by the ability of singlet oxygen generation, as the singlet oxygen yield of compound **PS5** was 2.5‐fold compared to *m*‐THPC. In addition, the efficacy of chlorin photosensitizers could also be affected by the administration of drugs and the intracellular uptake in the organism.^38^ To gain additional information on the origins of the very high PDT efficacy of **PS5**, a pharmacokinetic study on plasma concentration of chlorophyll derivatives was carried out and complemented by molecular dynamics simulations of the permeation of the drug through a lipid bilayer.

#### Plasma Concentration of Chlorophyll Derivatives

**Figure 9:**
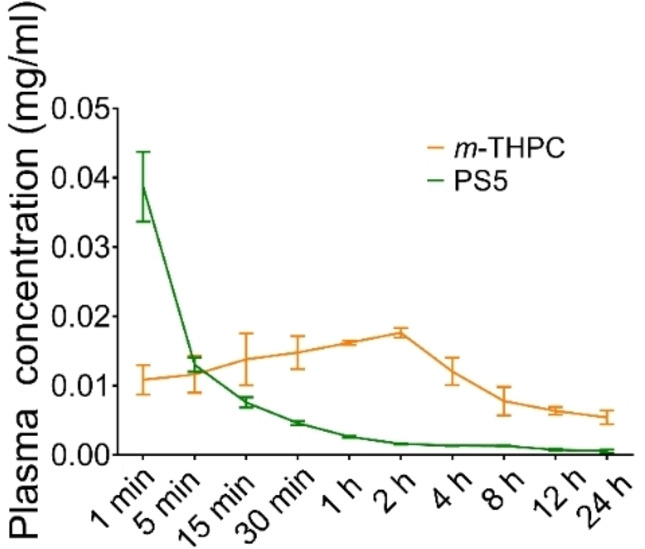
The plasma concentration of compounds (*m*‐THPC and **PS5**)

Photobleaching is one of the important factors affecting the photodynamic effect. Blood samples were collected from the mice after a tail intravenous injection of the compound, then plasma was separated and the intensity of the solution was measured by a fluorescence spectrophotometer. It was found that the plasma concentration of **PS5** in mice reached the maximum value at 1 min after administration (Figure 9). As time went on, the plasma concentration decreased. We found compound **PS5** metabolizes more rapidly in the blood than *m*‐THPC^39^ with significantly higher drug levels for the first 3 minutes, and smaller than *m*‐THPC afterwards. The high concentration of **PS5** in plasma proteins at early times indicates a high efficiency of drug transport in vivo. Thus, the treatment with **PS5** will show positive efficacy earlier than *m*‐THPC. The rapid entry of **PS5** into the blood circulation (within 1 minute) makes this compound advantageous for intravenous drug administration. In addition, quick clearance of **PS5** in vivo can be very beneficial for reducing skin toxicity side effects.^37^

## Computational Study

### Photophysical Properties

Photobleaching is one of the important factors affecting the photodynamic effect. The experimental results are in good accord with the photophysical properties obtained by DFT and TDDFT simulations of absorption spectra and calculated electronic properties of the **PS5** and reference molecule *m*‐THPC. The inspection of the simulated absorption spectra plots (Supplementary information) indicates that in the investigated molecule the first excited state is responsible for observing the Q band in the therapeutic window between 500 and 700 nm. The leading configuration of this state is HOMO‐LUMO excitation with a weight of 0.64.

The energies of HOMO orbitals for *m*‐THPC and **PS5** are 5.36 and −5.41 eV, respectively (Table 3). Larger stabilization (>0.14 eV) of the LUMO energy of **PS5** with respect to the reference LUMO level in *m*‐THPC, leads to a smaller HOMO‐LUMO gap. Thus, the estimated Δ(*ϵ*_LUMO_ ‐ *ϵ*_HOMO_) value for *m*‐THPC (2.82 eV) is larger than that of **PS5** (2.63 eV), which is in accordance with the relative Q‐band positions in UV‐vis spectra. The vertical excitation energies of the first excited state (*E*_exc_(S_1_)) are shifted to lower energies in the spectra of **PS5** with respect to the *E*_exc_(S_1_) for *m*‐THPC. On the other hand, the oscillator strength for the S1 state in molecule **PS5** is increased with respect to the 0.1046 value calculated for the S_1_ state of molecule *m‐*THPC. Of particular interest is the comparison of calculated S_1_‐T_1_ gaps with a measured generation rate of ^1^O_2_ for photosensitizers since the literature establishes that a more narrow S_1_‐T_1_ gap boosts ROS yield.^38,39^ We found that S_1_‐T_1_ gaps are similar for *m*‐THPC and **PS5**. Therefore, a better generation of singlet oxygen probably comes from the fact that the S_1_ state of **PS5** with higher oscillatory strength is more readily available.

**Table 3:** Energies of HOMO (HO) and LUMO (LU) orbitals, HOMO/LUMO energy gap, vertical excitation energies of the first singlet excited state, its oscillator strength, weight of leading configuration and singlet‐triplet energy gap at the PBE0/6‐311+G(2d,p) level of theory.

Molecule	*Ɛ*_HO_/eV	*Ɛ*_LU_/eV	HO‐LU gap /eV	*E*_exc_(S_1_) /eV
m‐THPC	‐5.37	‐2.55	2.82	2.211
PS5	‐5.41	‐2.78	2.63	2.128
	*f*(S_1_)	Weight	S_1_‐T_1_ gap / eV	
m‐THPC	0.1046	0.6067	0.913	
PS5	0.2073	0.6363	0.925	

### Membrane Permeability

**Figure 10:**
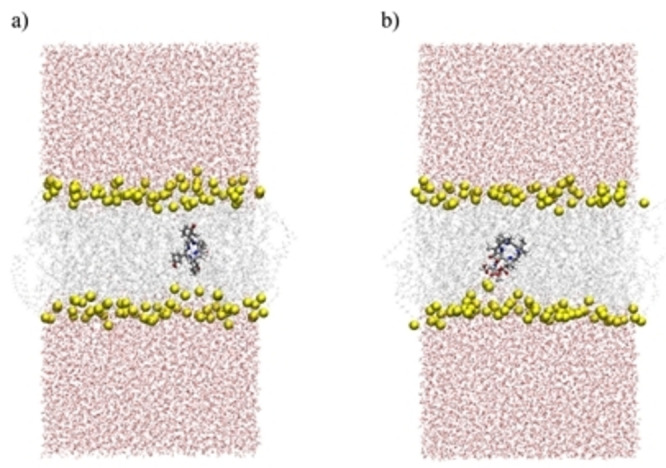
System setup for a) *m*‐THPC and b) **PS5** (snapshots taken from umbrella sampling simulation, window z=0, i. e., compounds located in the center of the POPC bilayer. Water is shown in licorice representation, with oxygen atoms colored red, POPC is denoted in grey (ghost representation), while phosphorus atoms belonging to lipid heads are depicted as yellow spheres. The compounds *m*‐THPC and **PS5** are shown in licorice representation. The systems are visualized using VMD.^45^

The molecular dynamics simulations of the permeation of **PS5** and *m*‐THPC through a model membrane^43,44^ have been performed to identify the nature of the intermolecular interactions in the bilayer and differences in their cellular uptake. The behaviour of the compounds in the membrane environment was investigated in the system containing solvated POPC bilayers and a single molecule of either **PS5** or *m*‐THPC (Figure 10).

**Figure 11:**
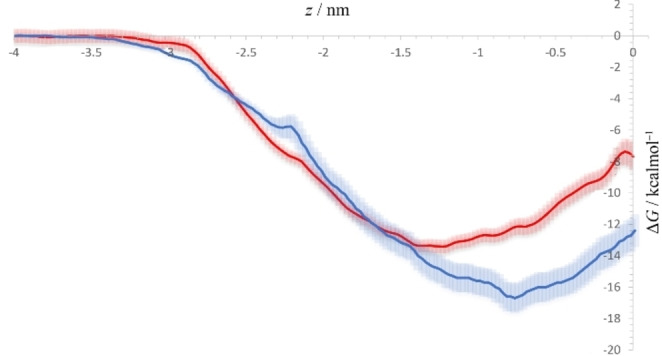
Free energy profiles for *m*‐THPC (blue) and **PS5** (red) in the POPC bilayer, with the profiles being set at 0 in the bulk water. Position‐dependent errors in the free energy obtained via bootstrapping (see Computational Methodology) are denoted with transparent blue/red shaded surfaces in the case of *m*‐THPC and **PS5**, respectively.

The analysis of free energy profiles for both investigated compounds shows that both *m*‐THPC and **PS5** are strongly lipophilic, with both possessing deep minimum in their free energy profiles inside the POPC bilayer, with *m*‐THPC possessing the deeper and narrower minimum of the two (−16.4 kcal mol^−1^ vs −13.6 kcal mol^−1^ for *m‐*THPC and **PS5**, respectively, Figure 11 . More precisely, *m*‐THPC tends to reside closer to the bilayer center, with its free energy minimum (ΔG) being located at approximately 0.8 nm from the bilayer center, with the same being found at ca. 1.3 nm from the center of the bilayer in the case of **PS5**, with its free energy minimum in the free energy profile being somewhat shallower compared to the equivalent *m*‐THPC′ feature. Both compounds possess a free energy maximum at the very center of the bilayer, with these maxima lying approximately 4 kcal mol^−1^ higher compared to their respective global minima in free energy profiles (Figure 11). Finally, neither compound shows a barrier when entering (Figure 11) from the water to the lipophilic phase, i. e., the entrance to the bilayer from water is spontaneous. The permeabilities of the two compounds were also evaluated by the inhomogeneous solubility diffusion permeability model.^46^ The position‐specific diffusion coefficients (Figure S16) were calculated and the permeabilities across the bilayer are 3⋅10^−10^ cm s^−1^ and 1⋅10^−8^ cm s^−1^ for *m‐*THPC and **PS5**, respectively. The low permeabilities for both compounds obtained via methodology assuming concentration equilibrium reflect the fact that both m‐THPC and **PS5** possess deep maxima in the free energy profile, thus possessing high ΔG^≠^, i. e., barrier height when passing from the bilayer toward the water phase. This naturally does not imply that the permeabilities of the two compounds would be as low in the non‐equilibrium, i. e., in the concentration‐gradient regime. The calculated difference in the permeability coefficients for m‐THPC and **PS5** (later has a higher coefficient), indicates an increased permeability of **PS5** through the membrane.^47^

However, the difference in diffusion properties between **PS5** and *m*‐THPC is not the only factor contributing to better **PS5** activity. First, the difference between the diffusion coefficients is not very large, which is in accordance with the data shown in Figure 7. Specifically, it is evident that the antitumor activity without light is similar for both compounds. However, once the cells are irradiated with the laser light, the activity of **PS5** is significantly increased.

## Experimental

### Chemistry Methods. General Information

Spirulina Pacifica algae powder (All Natural) was used to extract chlorophyll from blue‐green algae. Commercially available reagents (Aldrich, Fluka, Merck) were used without additional purification. All reactions were carried out in dry solvents. A standard method was used to dry toluene over sodium and distilled. Silica gel (Silica gel 60, 70–230 mesh, Fluka) was used for column chromatography. Organic solvents were removed on a rotary evaporator at reduced pressure (10‐20 mm Hg) (Büchi). Thin layer chromatography (TLC) (silica gel 60 F_254_, Merck) was used to monitor reactions and sample purification by chromatography. The prepared compounds were structurally characterized by ^1^H NMR spectroscopy using Bruker Avance 300 MHz NMR or Bruker Avance 600 NMR spectrometers. Commercially available deuterated chloroform was used for NMR. The chemical shifts (δH and δC) are expressed against tetramethylsilane (TMS) as an internal standard. Multiplicity designations: s=singlet, d=doublet, t=triplet, q=quartet, m=multiplet, bs=broad signal. UV‐vis spectrophotometric measurements were performed on a Varian Cary 100 Bio spectrophotometer in the wavelength range from 200 to 800 nm. Infrared Spectra (FTIR‐ATR) were recorded on a Fourier Transform Infrared Attenuated Total Reflection PerkinElmer UATR Two spectrometer in the range from 400 to 4000 cm^−1^. The exact mass was determined on a 4800 MALDI TOF/TOF Analyzer (Applied Biosystems) mass spectrometer using positive ionization at the Center for Proteomics and Mass Spectrometry, Ruđer Bošković Institute. A small amount of sample was mixed with the CHCA matrix and applied to the MALDI plate. The purity of the compound **PS5** was ascertained by HPLC analysis on the Varian ProStar 230 HLPC instrument. This compound was >95 % pure.

### Materials

Dimethyl pheophorbide a (**6**) has been prepared from Spirulina Pacifica according to known procedure.^23^

### Trimethyl‐15^2^‐[L‐aspartyl] Pheophorbide a (5).

Dimethyl pheophorbide *a* **6** (50 mg, 0.083 mmol) was dissolved in dry toluene (2 mL) and the solution was transferred to a thick‐wall glass vessel with a vacuum Teflon tap. Dimethyl L‐aspartate hydrochloride (40 mg, 0.202 mmol) and triethylamine (1 drop) were added to the solution. The high‐pressure vessel was sealed and the reaction mixture was heated for 2 hours at 110 °C in an oil bath. After cooling to room temperature, the solvent was removed in high vacuum, and the residue was subjected to flash column chromatography on silica gel and eluted with CH_2_Cl_2_: acetone 95 : 5 mixture. The purified product was chromatographed again on column (silica gel, CH_2_Cl_2_: acetone 95 : 5), and the product was obtained as a black solid (54 mg, 90 % yield). ^1^H NMR (300 MHz, CDCl_3_) δ/ppm: 9.51 (1H, s, H10), 9.38 (1H, s, H5), 8.57 (1H, s, H20), 7.99 (1H, dd, *J*=18.8, 11.2 Hz, H3^1^), 7.85 (1H, d, *J*=9.0 Hz, NH), 6.28 (1H, dd, *J*=18.8, 1.4 Hz, H3^2^), 6.17 (1H, s, H15^1^), 6.15 (1H, dd, *J*=11.2, 1.4 Hz, H3^2^), 5.15 (1H, dd, *J*=8.2, 4.9 Hz, H15^3^), 4.53 (1H, td, *J*=8.9, 2.1 Hz, H17), 4.41 (1H, dq, *J*=15.1, 1.7 Hz, H18), 3.84 (3H, s, OCH_3_‐17), 3.74 (3H, s, CH_3_‐12), 3.71 (2H, q, *J*=7.8 Hz, H8^1^), 3.67 (3H, s, OCH_3_), 3.60 (3H, s, OCH_3_), 3.39 (3H, s, CH_3_‐2), 3.23 (3H, s, CH_3_‐7), 3.13 (1H, dd, *J*=16.9, 5.4 Hz, H15^4^), 2.99 (1H, dd, *J*=16.9, 5.4 Hz, H15^4^), 2.52‐2.66 (2H, m, H17^1^, H17^2^), 2.10‐2.32 (2H, m, H17^1^, H17^2^), 1.85 (3H, d, *J*=7.1 Hz, CH_3_‐18^1^), 1.69 (3H, t, *J*=7.8 Hz, CH_3_‐8^2^), 0.51 (1H, brs, NH), −1.61 (1H, brs, NH). ^13^C NMR (150 MHz, CDCl_3_) δ/ppm: 10.7, 11.6, 16.9, 18.9, 22.5, 27.9, 29.2, 29.5, 30.4, 35.9, 48.6, 49.9, 50.5, 51.2, 51.6, 52.2, 64.6, 92.7 (C3^1^), 96.9, 103.7, 122.4 (C3^2^), 128.1, 128.4, 128.7, 131.3, 135.6, 135.7, 135.9, 137.4, 141.4, 144.6, 149.4, 150.4, 155.0, 162.2, 167.1, 170.7, 170.8, 173.3, 190.8 (three C overlapped). UV/Vis (CDCl_3_), λ_max_ (nm): 418, 509, 541, 612, 672. IR (cm^−1^): 1732 (C=O), 1694 (C=O), 1672 (C=O). HRMS (MALDI): *m/z* calculated for C_41_H_45_O_8_N_5_+H^+^ [M+H]^+^: 736.3346, found: 736.3328.

### Computational Methodology

#### Photophysical Calculations

The structures of investigated molecules were optimized in the ground state using the B3LYP/6‐311+G(d) method.^48,49^ Vibrational analyses were performed to verify the nature of the energy minimum. In these calculations, ultrafine grid and Grimme's GD3 dispersion correction^50^ were used. The vertical excitation energies and their oscillator strengths for 40 singlet states and 40 triplet states were calculated utilizing the time‐dependent functional theory (TD‐DFT) using PBE0 density functional.^51^ The choice of functionals for this study was based on the recent review of the performance of various density functionals for calculating the excitation energies in different molecules.^52^ All single‐point vertical excited energy calculations have been performed by using a 6–311+G(2d,p) basis set. Simulation of absorption spectra from the calculated excitation energies and oscillator strengths was conducted by simple convolution fit with Gaussian functions with a half‐width of 0.4 eV by using an in‐house created Perl script written by Prof. Mario Barbatti.^53^ The DFT and TDDFT electronic data were obtained using the Gaussian09 program^54^ while Vega‐ZZ^55^ and Molden^56^ programs were used for visualization and geometry manipulations.

#### Membrane Simulations

To inspect the behavior of the two investigated compounds **PS5** and *m*‐THPC in a membrane environment, the system was constructed containing 64 POPC lipids per leaflet, ca. 12400 water molecules, a single molecule of either **PS5** or *m*‐THPC and 25 sodium and chloride ions, to emulate the physiological ionic strength of 0.1 mol dm^−3^ present in the conducted experiments. Molecular dynamics simulations were performed in a periodic box with the size of ca. 6.6 nm x 6.6 nm x 12.9 nm, in both cases. SLipids force field^57−59^ was used for POPC molecules and TIP3P^60^ for water. Parameters describing **PS5** and *m*‐THPC moieties were obtained in a manner consistent with the SLipids force field, namely via general Amber force field (GAFF)^61^, whereby the atomic charges of the two compounds were calculated using the usual GAFF protocol, i. e., by first conducting geometry optimization at B3LYP/6‐31G(d) level of theory, with the single point ESP charge calculation obtained at HF/6‐31G(d) level of theory. The RESP method^62^ was used to obtain the final sets of partial charges for both **PS5** and *m*‐THPC using the AMBER Antechamber module.^63^ All simulations were performed at a constant temperature of 310 K employing the Nosé‐Hoover thermostat^64^ independently for the POPC bilayer/compound and water/ions subsystems, with a coupling constant of 0.5 ps^−1^. Pressure was maintained at 1 bar using the semi‐isotropic Parrinello‐Rahman barostat^65^ with the time constant for pressure coupling being 10 ps^−1^. Electrostatics were obtained by the particle‐mesh Ewald (PME) method^66^ with the real space Coulomb interactions cut off at 1.2 nm using a Fourier spacing of 0.12 nm with the Verlet cut‐off scheme. Minimization and equilibration of the prepared systems, where the compound of interest (**PS5** or *m*‐THPC) was placed inside the water phase (bulk water), and whose position was restrained (center of mass of **PS5** or *m*‐THPC set at 4.5 nm from the bilayer center, harmonic force constant of 1000 kJ mol^−1^ nm^2^ used to keep the distance throughout minimization/equilibration stages), was performed using the common CHARMM‐GUI minimization/equilibration procedure.^67^ Initial configurations for umbrella sampling simulations were generated from a pulling simulation, where a single **PS5** or *m*‐THPC molecule was pulled from the bulk water toward the bilayer center, using a force constant of 1000 kJ mol^−1^ nm^2^ and rate of 0.001 nm ps^−1^. Thus, 45 initial configurations for each system (POPC/**PS5** and POPC/*m*‐THPC) were prepared, spanning distances from −0.1 to 4.3 nm from the bilayer center in the z direction, thereby covering more than half of the entire bilayer width. A harmonic restraint with a force constant of 1000 kJ mol^−1^ nm^2^ was applied to the distance between the centre of mass of **PS5** or *m‐*THPC and the centre of mass of the bilayer with 0.1 nm spacing between the two neighbouring windows. In the case of both compounds, the simulation time of 100 ns per umbrella window was used with a 2 fs time step. The first 20 ns were considered as equilibration in each window and were discarded from further analysis. Final free energy curves were obtained using the WHAM procedure to obtain the profiles, while the error bar was calculated using the Bayesian bootstrap analysis with 100 bootstraps.^68^

#### Permeability

Theoretically, the inhomogeneous solubility diffusion permeability model^69^ can be used to obtain the permeability coefficient of a molecule in an atomistic simulation‐based PMF approach, with the relation used to obtain permeability being 1

$$\frac{1}{P}=R=\underset{z1}{\int^{z2}}\frac{e^{\beta [G(z)]}}{D\left(z\right)}dz,$$

with *R* being the overall resistivity of the membrane/water toward the compound of interest in the domain with the boundaries z1 and z2, *G*(*z*) is the calculated free energy profile, β is 1/*k*_B_T, while *D*(*z*) is the position‐specific diffusion coefficient, which can be calculated via Hummer's positional autocorrelation extension of Wolf‐Roux estimator^70^ with the autocorrelations being obtainable from the trajectories of umbrella sampling simulations. We thus calculated the position‐specific diffusion coefficients for both *m*‐THPC and **PS5**. Permeability is then calculated according to the above equation, with z1=0 nm, and z2=3.5 nm, describing permeability through one‐half of the bilayer. Final permeability is then obtained by dividing the half‐width result by 2, due to the underlying assumption that the free energy profile is symmetric with respect to z=0.

## Conclusions

In summary, a novel chlorophyll derivative, trimethyl‐15^2^‐[L‐aspartyl]pheophorbide a (**PS5**) had been designed and synthesized. The compound **PS5** had strong absorption and fluorescence emission in the phototherapeutic window. Photosensitizer **PS5** was stable and had a relatively high ^1^O_2_ quantum yield under 650 nm laser irradiation. **PS5**‐PDT effectively reduced the cell viability in a drug dose‐dependent and light dose‐dependent manner. It could inhibit the growth of A549 cells after PDT in vitro. It localizes in mitochondria, the lysosomes, and the endoplasmic reticulum. In vivo **PS5** showed an obvious photodynamic anti‐tumor effect. Molecular dynamics calculations are in good accordance with the experiment supporting increased membrane permeability for **PS5**, compared to *m‐*THPC. TDDFT calculations indicate even more pronounced singlet oxygen generation in **PS5**, due to a lower HOMO‐LUMO gap and a more readily available S_1_ excited state. These results indicated that **PS5** is a promising photosensitizer worthy of further pre‐clinical evaluation.

## Author Contributions

A.B., and A.K. carried out the synthetic chemistry. D.‐Y.C. carried out a photophysical study. Y.‐H.G. carried out the studies of cytotoxicity of compounds, intracellular localization, caspase activity assay, in vivo PDT efficacy and plasma concentration. Z.B., and M.V. carried out molecular dynamic simulations, while I.A. performed quantum chemical calculations. Y.‐J.Y., Z.‐L.C., and D.M. conceived the study. Y.‐H.G., Z.‐L.C., and D.M. secured the funding. M.V., Z.‐L.C., Y.‐J.Y., Y.‐H.G., and D.M. prepared the manuscript.

## Conflict of Interests

The authors declare no conflict of interest.

1

## Supporting information

As a service to our authors and readers, this journal provides supporting information supplied by the authors. Such materials are peer reviewed and may be re‐organized for online delivery, but are not copy‐edited or typeset. Technical support issues arising from supporting information (other than missing files) should be addressed to the authors.

Supporting Information

## Data Availability

The data that support the findings of this study are available in the supplementary material of this article.
