# Supplementary material for: Significant Tumor Inhibition of Trimethyl‐152‐[L‐aspartyl]pheophorbide a in Tumor Photodynamic Therapy≠
Source: ChemMedChem. 2025 Apr 11;20(10):e202500087. doi: 10.1002/cmdc.202500087 (PMC12091846; doi:10.1002/cmdc.202500087)
Supplement: Supplementary file 1 — Supporting Information [file CMDC-20-e202500087-s001.pdf]

ChemMedChem

Supporting Information

Significant Tumor Inhibition of Trimethyl-15^2-[L-aspartyl]pheophorbide a in Tumor Photodynamic Therapy[‡]

Anita Benić, Akmaral Kussayeva, Ivana Antol, Mario Vazdar, Zlatko Brkljača, Dan-Ye Chen, Yi-Jia Yan, Ying-Hua Gao,* Zhi-Long Chen,* and Davor Margetić*

Supporting Information

Significant Tumor Inhibition of Trimethyl-15^2-[L-aspartyl]pheophorbide a in Tumor Photodynamic Therapy

Anita Benić, Akmaral Kussayeva, Ivana Antol, Vazdar Mario, Brkljača Zlatko, Dan-Ye Chen, Yi-Jia Yan, Ying-Hua Gao*, Zhi-Long Chen*, and Davor Margetić*

Contents:

Figures S1-S2. ^{1}H and ^{13}C NMR spectra of compound **PS5**. Page S2
Figure S3. COSY NMR spectra of compound **PS5**. Page S3
Figure S4. COSY NMR spectra of compound **PS5**. Page S4
Figure S5. UV-vis spectra of compound **PS5**. Page S5
Figure S6. IR spectra of compound **PS5**. Page S5
Figures S7-S8. HRMS spectra data of compound **PS5**. Page S6
Figure S9. HPLC chromatogram of compound **PS5**. Page S7
Details on measurements of photophysical properties. Page S7
Method for singlet oxygen determination. Page S7
Biological evaluation.
 Chemicals and reagents. Page S8
 Cell culture. Page S8
 MTT assay. Page S8
 Subcellular localization assay. Page S8
 Caspase 3 activity. Page S9
 Animal models. Page S9
 In vivo therapeutic efficacy. Page S9
Figure S10. The body weight of A549 xenograft tumor mice Page S9
Details on statistical analysis. Page S10
Intracellular localization Page S10
Plasma concentration of chlorophyll derivatives Page S10
Computation of photophysical properties Page S11
Figure S11. Optimized structures of *m*-THP, and **PS5** Page S11
Figure S12. Simulated UV-Vis spectra of *m*-**THPC** Page S11
Table ST1. TD-DFT calculated el. properties for *m*-**THPC** Page S11
Figure S13. Simulated UV-Vis spectra of **PS5** Page S14
Table ST2. TD-DFT calculated el. properties for **PS5** Page S14
Figure S14. Comparison of the simulated UV/Vis spectra Page S16
Figure S15. HOMO and LUMO Kohn-Sham orbitals Page S17
Figure S16. Position-specific diffusion coefficients Page S17
Cartesian coordinates for optimized structures Page S18

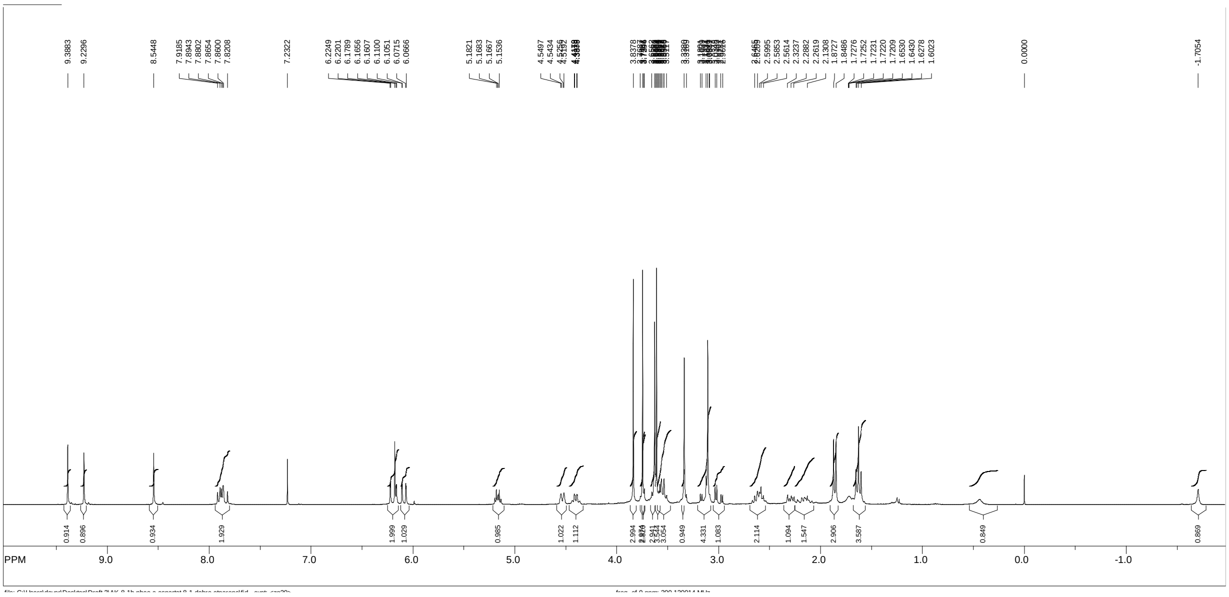

Figure S1. ^{1}H NMR spectrum of trimethyl 15^{2}-L-aspartyl] pheophorbide a (**PS5**) in $CDCl_3$ (300 MHz)

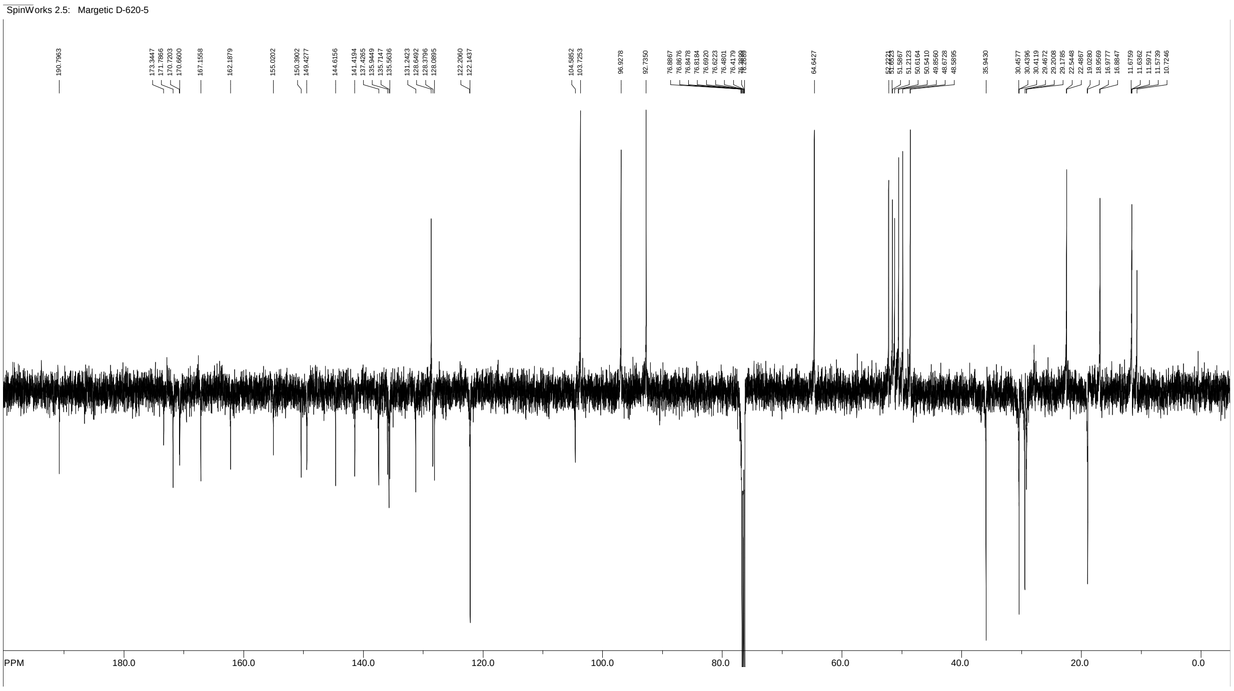

Figure S2. ^{13}C NMR spectrum of trimethyl 15^{2}-[L-aspartyl] pheophorbide a (**PS5**) in $CDCl_3$ (300 MHz)

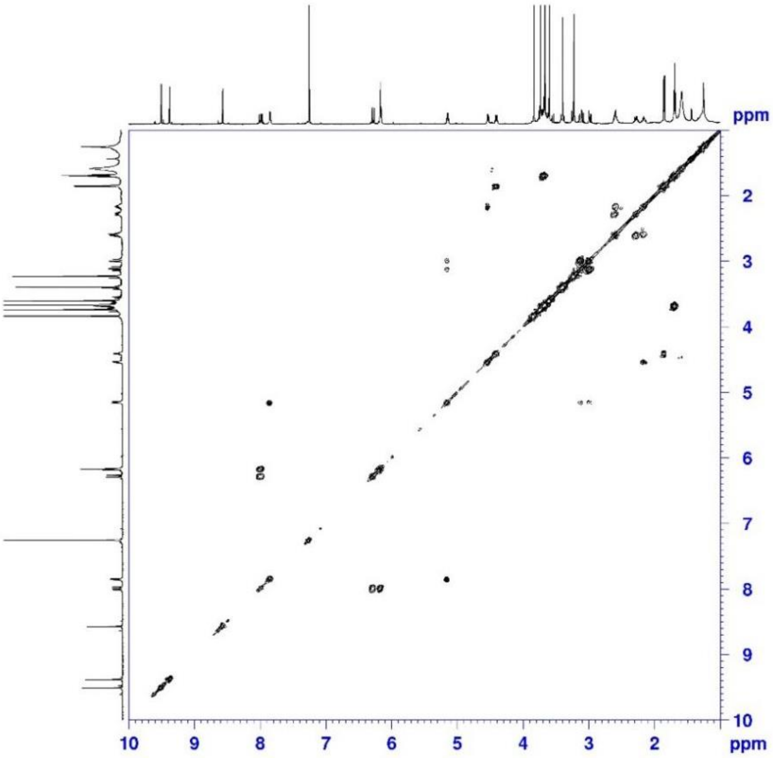

Figure S3. COSY NMR spectrum of trimethyl 15[2]-[L-aspartyl] pheophorbide a (**PS5**) in $CDCl_3$ (600 MHz)

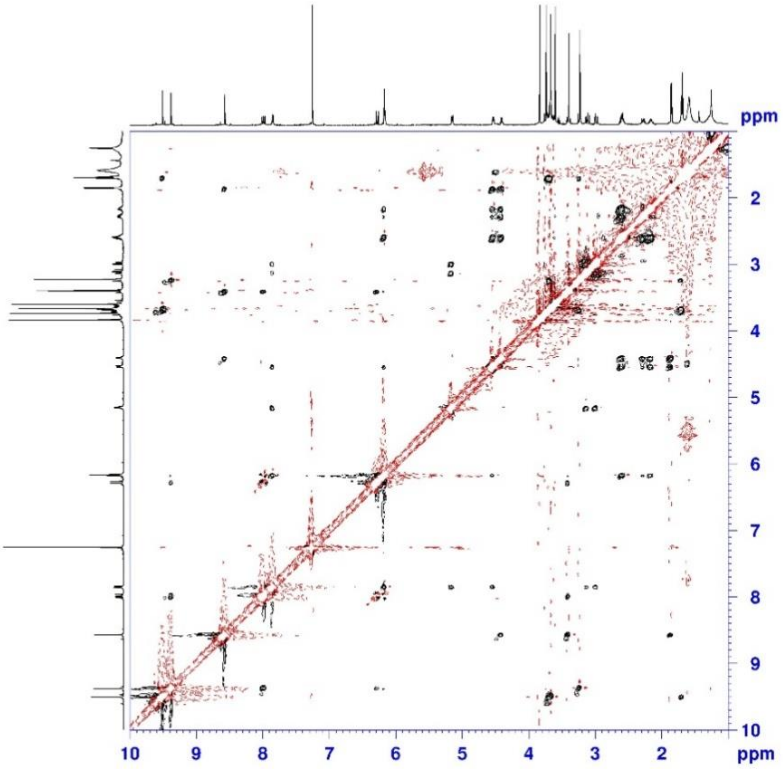

Figure S4. NOESY NMR spectrum of trimethyl 15^2-[L-aspartyl] pheophorbide a (**PS5**) in $CDCl_3$ (600 MHz)

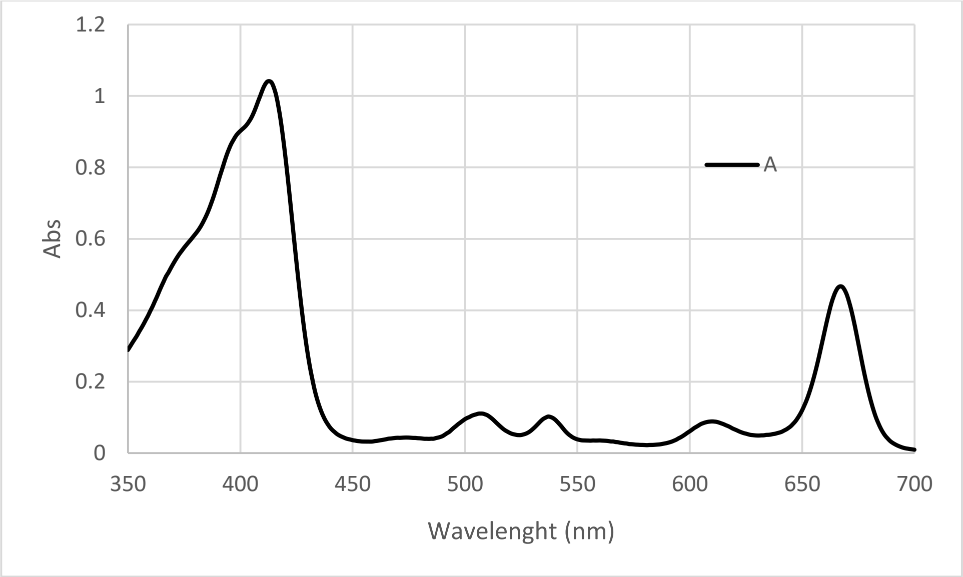

Figure S5. UV-Vis spectrum of trimethyl 15^2-[L-aspartyl] pheophorbide a (**PS5**) in $CHCl_3$

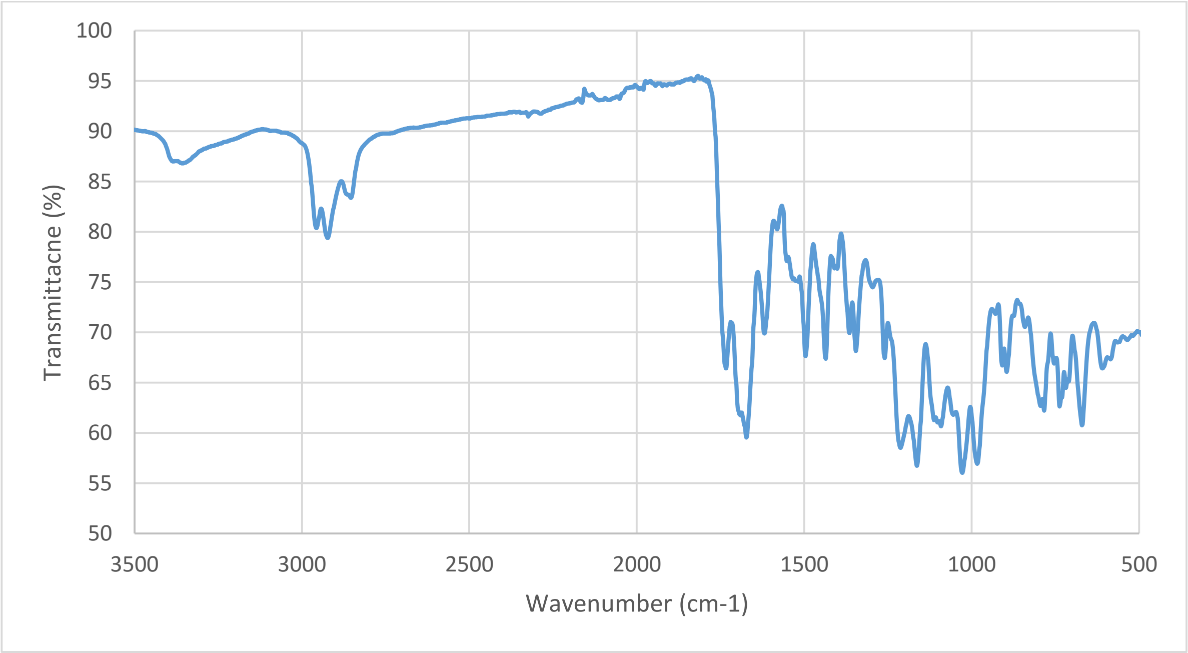

Figure S6. IR spectrum of trimethyl 15^2-[L-aspartyl] pheophorbide a (**PS5**)

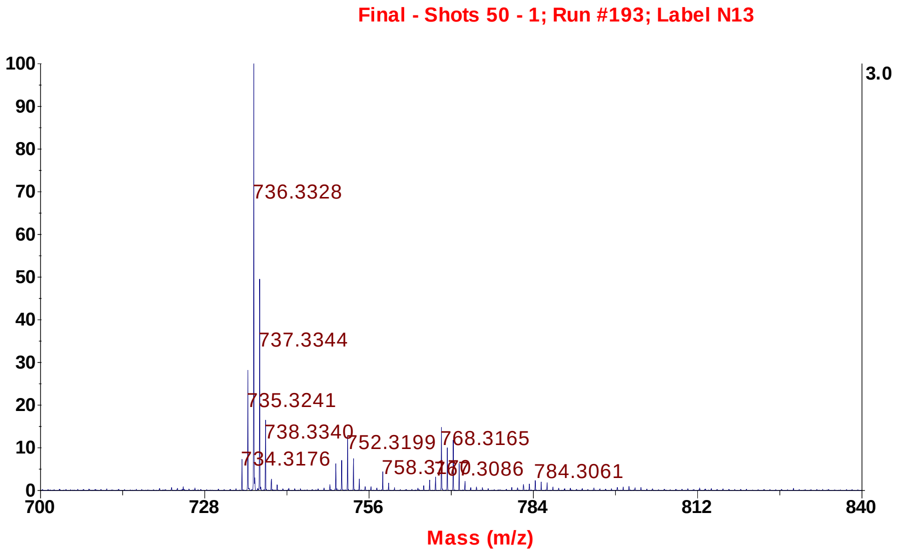

Figure S7. Mass spectrum of trimethyl 15^2-[L-aspartyl] pheophorbide a (**PS5**) in the range *m/z* 700-840

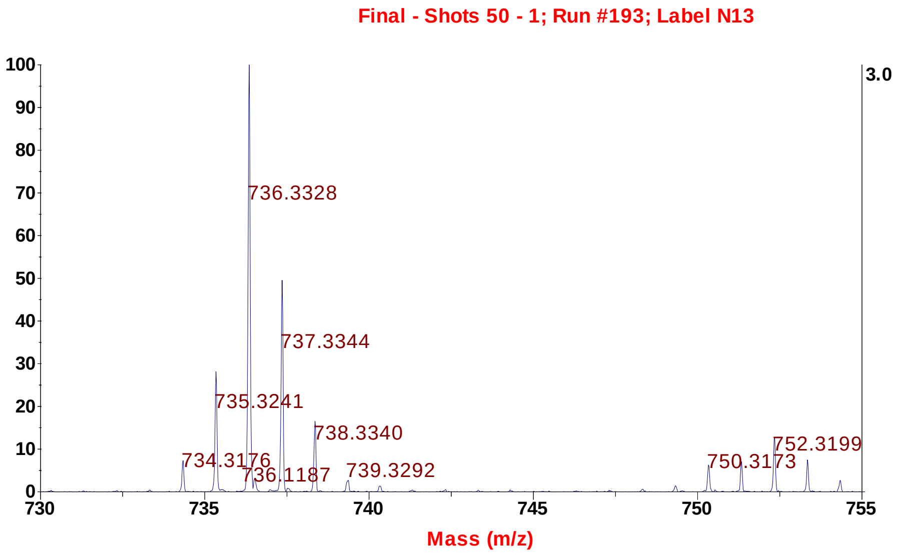

Figure S8. Mass spectrum of trimethyl 15^2-[L-aspartyl] pheophorbide a (**PS5**) in the range *m/z* 730-756

Analytical HPLC method: Varian ProStar 230 HLPC instrument with Microsorb-MV 100-5 column (C18, 150 mm × 4.6 mm x 1/4 inch , 5 µm); solvent system: A) MeOH : NH_4OAc (0.5 N in water); B) acetone : MeOH = 80 : 20. Program: 1) 0 - 27 min (80% -5 % A), 2) 27-34 min (5 % A) 3) 34-36 min (5 % - 80 % A). Flow rate 1.5 mL/min; wavelength 414 nm; injection volume 10 µL.

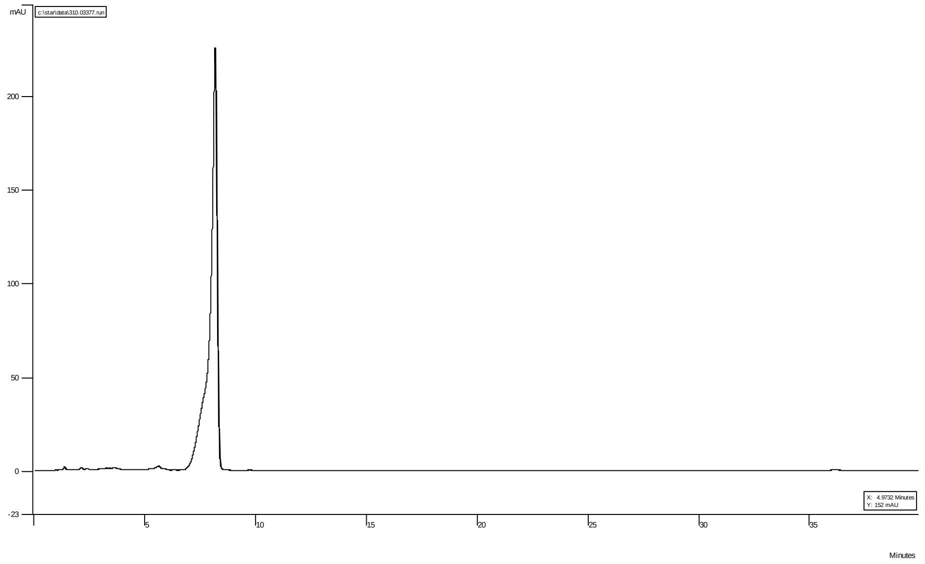

Figure S9. HPLC spectrum of compound **PS5** (t_R = 8.16 min).

PHOTOPHYSICAL PROPERTIES

Photophysical properties

UV-vis absorption spectrum was measured on an Ultraviolet-visible Spectrophotometer (Model V-530, Japan). Fluorescence measurements were carried out with a Fluorescence Spectrometer (FluoroMax-4, France). Compound **PS5** was dissolved in DMSO to get the 10 µM concentration solutions. Slits were kept narrow to 1 nm in excitation and 1 nm in emission. All the measurements were carried out at room temperature.

Evaluation of singlet oxygen generation

In vitro photo-bleaching was conducted by observing the UV-vis spectra of compound **PS5** at 10 µM concentrations dissolved in DMSO. The solution of compound **PS5** in the cuvette was then irradiated with a 650 nm laser (therapeutic light) at various time points for 90 min. The UV-vis spectra were obtained every 10 min in 90 min.

The $^1O_2$ generation was evaluated with DPBF as a singlet oxygen ($^1O_2$) scavenger.[1] A mixed solution of compound (2 µM) and DPBF (60 µM) was irradiated with a 650 nm laser and the

absorption value at 417 nm was monitored using a UV-vis spectrophotometer every 10 s. The observed rate constants (k) were calculated from ln ($[DPBF]_t$ / $[DPBF]_0$) = - kt, where $[DPBF]_t$ and $[DPBF]_0$ represent the ultraviolet-visible absorbance at time t and time 0, respectively.[2] Comparison of the slopes for the samples with the reference yielded the singlet oxygen quantum yield straightforwardly as given in the equation below.

$$\Phi_{\Delta}^{S} = \frac{K_S}{K_R} \times \Phi_{\Delta}^{R}$$

The superscripts/subscripts S and R stand for the sample and the reference, respectively.

BIOLOGICAL EVALUATION

Chemicals and reagents

3-(4,5-Dimethylthiazol-2-yl)-2,5-diphenyl tetrazolium bromide (MTT), the Hoechst 33342, Mito-Tracker Green (MTG), Lyso-Tracker Blue and ER-Green [DiOC6(3)] were purchased from KeyGen BioTech, China. 1,3-diphenylisobenzofuran (DPBF), dimethyl sulfoxide (DMSO), and *N*, *N*-dimethylformamide (DMF) were obtained from Sinopharm Chemical Reagent Co., Ltd. All the chemicals and reagents were of analytical grade and used without any purification.

Cell culture

The lung cancer cells (A549) were purchased from the Type Culture Collection, the Chinese Academy of Sciences. The cell line was cultured in F-12K medium added with 10% heat-inactivated fetal bovine serum, 100 units/mL penicillin, 100 µg/mL streptomycin, and 1 mM glutamine. Cells were incubated at 37 °C in a humidified 5% CO_2 atmosphere.

MTT assay

The cells (4×10^5 cells/well) were seeded in 96-well micro-plates in a complete medium for dark and light-dependent cytotoxicity measurement. After 24 h, the cells were washed once and incubated for another 24 h with a complete medium containing various concentrations of compound (0, 0.1, 0.3, 0.5, 0.7, and 0.9 µM), respectively. All samples were handled in the dark. Cellular survival was measured using a 3-(4,5-dimethyl-2-thiazolyl)-2,5-diphenyl-2-*H*-tetrazolium bromide (MTT) assay. Briefly, at the end of the incubation period, 20 µl MTT (5 mg/mL) reagent was added to each well and incubated for 4 h at 37°C. Then, the medium was removed, and the formazan complex was solubilized with 150 µL DMSO. The absorbance of the complex was measured with a microplate reader at a wavelength of 570 nm.

Subcellular localisation assay

The cells were grown in 35 mm Petri dish and incubated at 37 °C with 5 μM **PS5** for 4 h in the dark, then rinsed in the medium and incubated with Mito-Tracker-Green (200 nM), Lyso-Tracker Blue (5 μM), or ER-Tracker Green (500 nM), respectively. After washed with PBS, the cells were fixed for 10 min at 4 °C with 4% paraformaldehyde and then examined by fluorescence with confocal microscopy (Carl Zeiss LSM 700, Jena, Germany). Photosensitizer **PS5** was excited at 415 nm and monitored at 600-700 nm.

ROS generation

A549 cells were seeded in a 35 mm cell culture dish at a density of 1 × 10^4 cells/well. After 24 h, the cells were treated with PS5. After 4 h, the cells were treated with PDT (650 nm) and stained with DCFH-DA according to the manufacturer's instructions.

Caspase 3 activity

Caspase activity was determined by a colorimetric assay based on the ability of caspase-3 to change acetyl-Asp-Glu-Val-Asp *p*-nitroanilide (Ac-DEVD-pNA) into a yellow formazan product (*p*-nitroaniline (pNA)). An increase in absorbance at 405 nm was used to quantify the activation of caspase activity.

Animal models

Balb/c nude mice (5 weeks old), were used to establish a lung cancer mouse model. Tumors were allowed to grow to about 150-170 mm^3 in volume before being used for PDT.

***In vivo* therapeutic efficacy**

The A549 tumor-bearing Balb/c nude mice were randomly divided into 5 groups: (1) PBS as a control, (2) 0.15 mg/kg *m*-THPC without irradiation, (3) 0.15 mg/kg **PS5** without irradiation, (4) 0.15 mg/kg *m*-THPC with irradiation, and (5) 0.15 mg/kg **PS5** with irradiation. Therapy was continued through tail vein injection for 14 days. The mice were locally irradiated with a 650 nm laser (200 mW/cm^2, 120 J/cm^2) after injection for PDT treatment. The body weight and the tumor size of each mouse were recorded to evaluate the therapy efficiency and toxicity: the tumor volume (V) was calculated: $V = a \times b^2/2$ (a, the length of the tumor; b, the width of the tumor). Finally, organs of the mice, including the heart, livers, spleen, lung, kidney, and tumors, were harvested and collected immediately for H&E and TUNEL histology analysis.

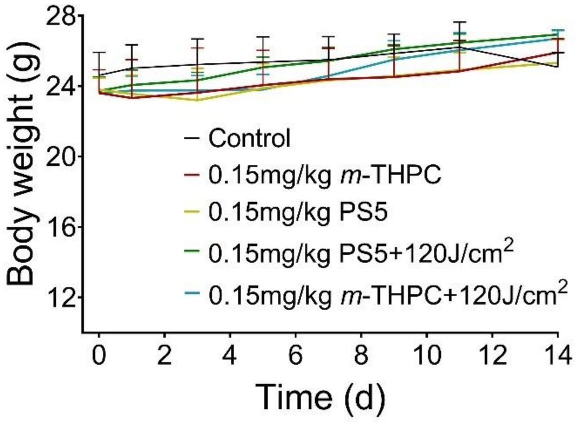

Figure S10. The body weight of A549 xenograft tumor mice with different treatments.

Statistical analysis

Graphics were done by Origin Pro 8.0 (Graph Software, USA). All results are presented as mean ± SD. Statistical significance was determined by unpaired two-tailed t-tests or two-way analysis of variance. * $P < 0.05$ and ** $P < 0.01$ were considered statistically significant.

Intracellular localization

The subcellular localization of photosensitizer **PS5** was evaluated by a confocal laser scanning microscope (CLSM). The experiments were conducted using the Mito-Track Green (MTG), Lyso-Track Green (LTG), and ER-Tracker Green (ETG).

Plasma concentration of chlorophyll derivatives

1. Drug dissolution

The compounds (**PS5**, *m*-THPC, 7.5 mg) were dissolved in dichloromethane (DCM, 3 mL) and then polyoxyethylene castor oil (200 μL) was added. After mixing, the organic solvent was evaporated by a rotary evaporator. Finally, the samples with PBS were mixed to the final concentration. We found that compound **PS5** precipitates quickly when dissolute in PBS. *m*-THPC was dissolved in THF by the same method.

2. Standard curve

36 KM mice were randomly divided into groups (n=18): *m*-THPC, **PS5**. The mice were sacrificed after taking a blood sample. The photosensitizers were diluted in different concentrations (0, 0.00125, 0.0025, 0.005, 0.01, 0.015 mg/mL) with plasma. The samples were separated by centrifugation at 12000 rpm for 10 min at 4 °C. The supernatant (250 μL) was mixed with DMSO (2.75 mL) and the fluorescence intensity of the solution was measured by a fluorescence spectrophotometer.

3. Experiment Method

60 KM mice (28 - 30 g, males) were fed in the environment under constant temperature (25-26 °C) and humidity (50-60%), with a 12 h light/dark cycle. The mice were randomly divided into groups (n=30): *m*-THPC, **PS5**. The mice were sacrificed at different times after a tail vein

intravenous injection of compounds (5 mg/kg). Blood samples were collected into the polypropylene microfuge tubes containing heparin sodium. Plasma was separated by centrifugation at 12000 rpm for 10 min at 4 ^{o}C. Plasma (250 μL) was mixed with DMSO (2.75 mL) and the fluorescence intensity of the solution was measured by a fluorescence spectrophotometer.

COMPUTATION OF PHOTOPHYSICAL PROPERTIES

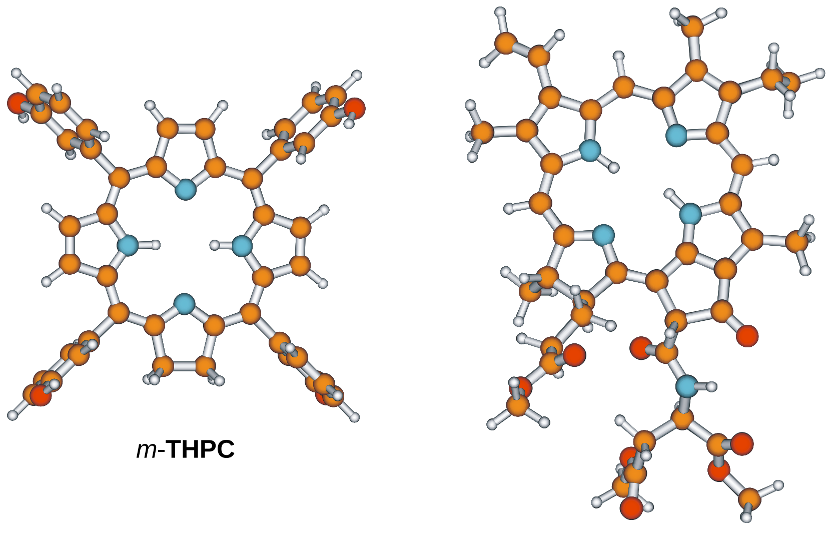

Figure S11. B3LYP/6-311+G(d) optimized structures of ***m*-THPC**, and **PS5**.

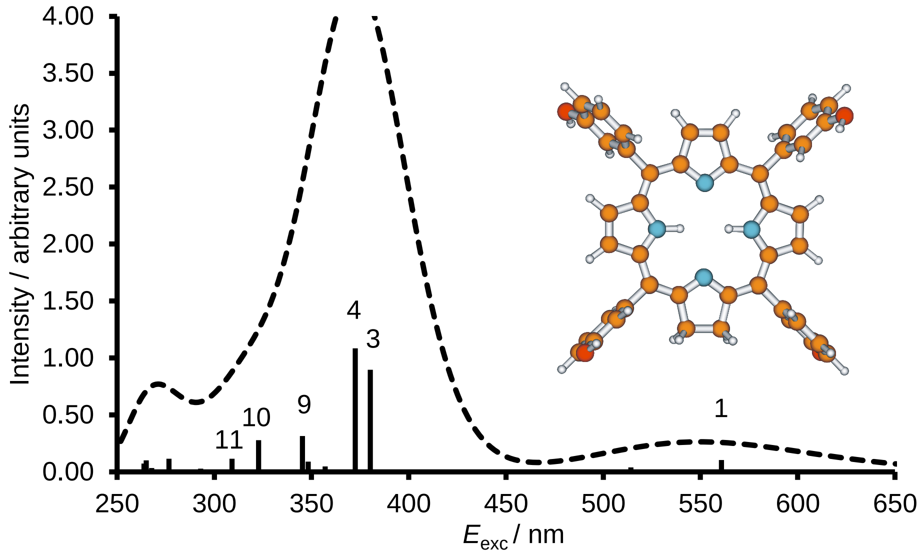

Figure S12. Simulated UV-Vis spectra of *m*-**THPC** calculated at TD DFT level of theory using PBE0 density functional and Pople type 6-311+G(2d,p) basis set.

Table ST1. TD-DFT calculated vertical excitation energies (E_{exc}), oscillator strengths (*f*) and leading configurations for *m*-**THPC** at the PBE0/6-311+G(2d,p) level of theory.

No.	E_{exc}/eV	*f*	Leading configurations		Coefficients	E_{exc}/nm
			Singlet excited states			
**1**	**2.211**	**0.1046**	**Singlet-A**	**178 -> 179**	**0.60665**	**561**
2	2.411	0.0399	Singlet-A	177 -> 179	0.5720	514
**3**	**3.261**	**0.8954**	**Singlet-A**	**177 -> 180**	**0.57159**	**380**
**4**	**3.328**	**1.0843**	**Singlet-A**	**178 -> 180**	**0.52174**	**373**
5	3.380	0.0001	Singlet-A	176 -> 179	0.66986	367
6	3.473	0.0481	Singlet-A	175 -> 179	0.68646	357
7	3.559	0.089	Singlet-A	173 -> 179	0.61905	348
8	3.563	0.0018	Singlet-A	174 -> 179	0.68234	348
**9**	**3.590**	**0.3157**	**Singlet-A**	**172 -> 179**	**0.58721**	**345**
**10**	**3.842**	**0.2774**	**Singlet-A**	**176 -> 180**	**0.55369**	**323**
**11**	**4.010**	**0.1156**	**Singlet-A**	**171 -> 179**	**0.44768**	**309**
12	4.020	0.0109	Singlet-A	175 -> 180	0.68968	308
13	4.046	0.0044	Singlet-A	171 -> 179	0.43172	306
14	4.099	0.0006	Singlet-A	164 -> 179	0.49894	302
15	4.129	0.0132	Singlet-A	170 -> 179	0.56644	300
16	4.134	0.0117	Singlet-A	178 -> 181	0.56078	300
17	4.143	0.0036	Singlet-A	169 -> 179	0.41068	299
18	4.160	0.0001	Singlet-A	174 -> 180	0.69596	298
19	4.163	0	Singlet-A	178 -> 182	0.58282	298
20	4.169	0.0013	Singlet-A	173 -> 180	0.65997	297
21	4.231	0.0272	Singlet-A	168 -> 179	0.55084	293
22	4.275	0.0026	Singlet-A	178 -> 183	0.64847	290
23	4.279	0.0013	Singlet-A	167 -> 179	0.45539	290
24	4.292	0.017	Singlet-A	166 -> 179	0.55482	289
25	4.295	0.0041	Singlet-A	178 -> 184	0.57367	289
26	4.318	0.02	Singlet-A	177 -> 181	0.48866	287
27	4.336	0.0018	Singlet-A	163 -> 179	0.63248	286
28	4.342	0.01	Singlet-A	177 -> 182	0.65418	286
29	4.436	0.0004	Singlet-A	177 -> 183	0.66111	279
30	4.466	0.0104	Singlet-A	177 -> 184	0.69067	278
31	4.481	0.1153	Singlet-A	165 -> 179	0.44625	277
32	4.531	0.0034	Singlet-A	164 -> 180	0.4038	274
33	4.547	0.0157	Singlet-A	178 -> 185	0.46831	273
34	4.631	0.0333	Singlet-A	171 -> 180	0.493	268
35	4.636	0.0069	Singlet-A	178 -> 187	0.56692	267
36	4.646	0.0198	Singlet-A	178 -> 186	0.59212	267
37	4.678	0.0998	Singlet-A	170 -> 180	0.43204	265
38	4.699	0.0716	Singlet-A	170 -> 180	0.49694	264
39	4.715	0.0191	Singlet-A	178 -> 188	0.55358	263
40	4.777	0.0002	Singlet-A	178 -> 190	0.5761	260

Triplet excited states						
1	1.298	0	Triplet-A	177 -> 179	0.6727	955
2	1.525	0	Triplet-A	178 -> 179	0.6991	813
3	2.018	0	Triplet-A	178 -> 180	0.65763	614
4	2.257	0	Triplet-A	177 -> 180	0.68731	549
5	2.990	0	Triplet-A	172 -> 179	0.56951	415
6	3.070	0	Triplet-A	172 -> 180	0.56173	404
7	3.326	0	Triplet-A	176 -> 179	0.52608	373
8	3.349	0	Triplet-A	175 -> 179	0.56416	370
9	3.438	0	Triplet-A	174 -> 179	0.42763	361
10	3.451	0	Triplet-A	173 -> 179	0.47878	359
11	3.474	0	Triplet-A	171 -> 179	0.32909	357
12	3.536	0	Triplet-A	166 -> 179	0.4144	351
13	3.551	0	Triplet-A	167 -> 179	0.34712	349
14	3.603	0	Triplet-A	170 -> 184	0.25371	344
15	3.621	0	Triplet-A	174 -> 179	0.26605	342
16	3.624	0	Triplet-A	173 -> 179	0.38138	342
17	3.639	0	Triplet-A	174 -> 179	0.37212	341
18	3.680	0	Triplet-A	178 -> 185	0.32865	337
19	3.871	0	Triplet-A	164 -> 179	0.49282	320
20	3.892	0	Triplet-A	176 -> 180	0.51008	319
21	3.904	0	Triplet-A	175 -> 180	0.41853	318
22	3.921	0	Triplet-A	175 -> 180	0.40989	316
23	3.994	0	Triplet-A	163 -> 179	0.64203	310
24	4.074	0	Triplet-A	178 -> 181	0.4842	304
25	4.079	0	Triplet-A	178 -> 182	0.30295	304
26	4.109	0	Triplet-A	177 -> 185	0.29375	302
27	4.113	0	Triplet-A	174 -> 180	0.3895	301
28	4.124	0	Triplet-A	178 -> 182	0.26029	301
29	4.127	0	Triplet-A	170 -> 179	0.57903	300
30	4.146	0	Triplet-A	178 -> 183	0.34384	299
31	4.151	0	Triplet-A	173 -> 180	0.39615	299
32	4.194	0	Triplet-A	174 -> 180	0.36166	296
33	4.200	0	Triplet-A	177 -> 181	0.28567	295
34	4.212	0	Triplet-A	169 -> 179	0.31903	294
35	4.245	0	Triplet-A	168 -> 179	0.61044	292
36	4.268	0	Triplet-A	169 -> 179	0.47623	291
37	4.299	0	Triplet-A	177 -> 181	0.50334	288
38	4.302	0	Triplet-A	178 -> 183	0.42318	288
39	4.330	0	Triplet-A	177 -> 182	0.44712	286
40	4.339	0	Triplet-A	177 -> 182	0.35431	286

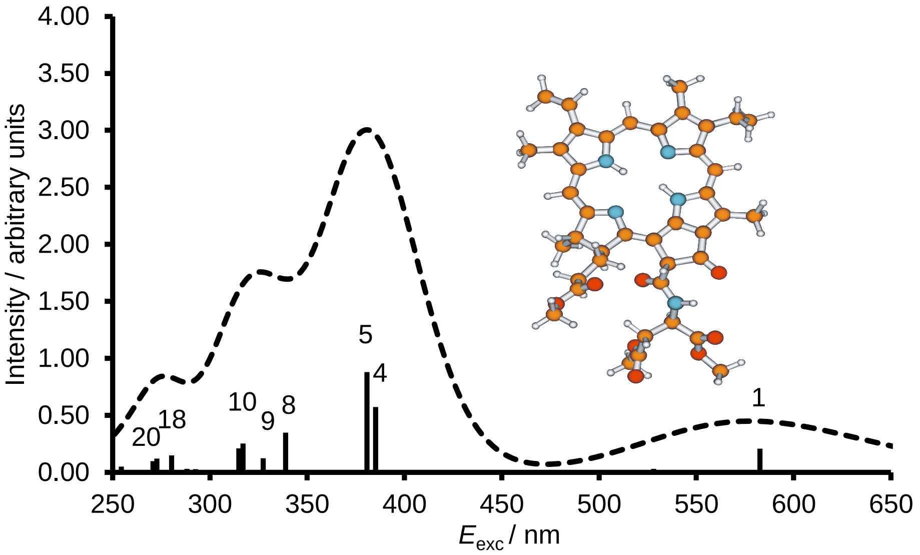

Figure S13. Simulated UV-Vis spectra of protonated guanidine derivative **PS5** calculated at TD DFT level of theory using PBE0 density functional and Pople type 6-311+G(2d,p) basis set.

Table ST2. TD-DFT calculated vertical excitation energies (E_{exc}), oscillator strengths (*f*) and leading configurations for **PS5** at the PBE0/6-311+G(2d,p) level of theory.

No.	E_{exc}/eV	*f*	Leading configurations		Coefficients	E_{exc}/nm
			Singlet excited states			
**1**	**2.128**	**0.2073**	**Singlet-A**	**195 -> 196**	**0.63629**	**583**
2	2.348	0.0306	Singlet-A	194 -> 196	0.59856	528
3	3.041	0.0090	Singlet-A	193 -> 196	0.68026	408
**4**	**3.219**	**0.5730**	**Singlet-A**	**195 -> 197**	**0.44638**	**385**
**5**	**3.257**	**0.8805**	**Singlet-A**	**194 -> 197**	**0.43976**	**381**
6	3.469	0.0088	Singlet-A	192 -> 196	0.69027	357
7	3.487	0.0017	Singlet-A	191 -> 196	0.60809	356
**8**	**3.659**	**0.3486**	**Singlet-A**	**193 -> 197**	**0.60047**	**339**
**9**	**3.787**	**0.1245**	**Singlet-A**	**195 -> 198**	**0.62899**	**327**
**10**	**3.912**	**0.2532**	**Singlet-A**	**194 -> 198**	**0.48578**	**317**
11	3.930	0.0728	Singlet-A	187 -> 196	0.57799	315
**12**	**3.937**	**0.2096**	**Singlet-A**	**190 -> 196**	**0.44452**	**315**
13	4.012	0.0011	Singlet-A	185 -> 196	0.53352	309
14	4.102	0.0106	Singlet-A	189 -> 196	0.62897	302
15	4.183	0.0067	Singlet-A	188 -> 196	0.63360	296
16	4.237	0.0292	Singlet-A	192 -> 197	0.61083	293
17	4.301	0.0312	Singlet-A	186 -> 196	0.47027	288
**18**	**4.423**	**0.1481**	**Singlet-A**	**195 -> 199**	**0.58789**	**280**
19	4.506	0.0046	Singlet-A	187 -> 197	0.45428	275
**20**	**4.545**	**0.1211**	**Singlet-A**	**194 -> 199**	**0.63704**	**273**
21	4.552	0.0031	Singlet-A	191 -> 197	0.51934	272
**22**	**4.578**	**0.0970**	**Singlet-A**	**184 -> 196**	**0.58886**	**271**

23	4.682	0.0020	Singlet-A	195 -> 200	0.63885	265
24	4.710	0.0059	Singlet-A	186 -> 197	0.38891	263
25	4.756	0.0069	Singlet-A	190 -> 197	0.47194	261
26	4.781	0.0009	Singlet-A	195 -> 201	0.55332	259
27	4.794	0.0120	Singlet-A	191 -> 198	0.42314	259
28	4.838	0.0003	Singlet-A	183 -> 196	0.67386	256
29	4.854	0.0061	Singlet-A	189 -> 197	0.42606	255
30	4.873	0.0515	Singlet-A	193 -> 198	0.59732	254
31	4.880	0.0074	Singlet-A	195 -> 202	0.57646	254
32	4.928	0.0046	Singlet-A	194 -> 200	0.59400	252
33	4.937	0.0012	Singlet-A	195 -> 203	0.49136	251
34	4.988	0.0004	Singlet-A	188 -> 197	0.63489	249
35	5.004	0.0004	Singlet-A	182 -> 196	0.68422	248
36	5.010	0.0046	Singlet-A	194 -> 201	0.48775	247
37	5.020	0.0280	Singlet-A	194 -> 201	0.37354	247
38	5.112	0.0019	Singlet-A	186 -> 197	0.46472	243
39	5.119	0.0096	Singlet-A	194 -> 202	0.46279	242
40	5.128	0.0004	Singlet-A	195 -> 205	0.34539	242
			Triplet excited states			
1	1.203	0	Triplet-A	194 -> 196	0.6257	1031
2	1.420	0	Triplet-A	195 -> 196	0.6594	873
3	2.039	0	Triplet-A	195 -> 197	0.6538	608
4	2.342	0	Triplet-A	194 -> 197	0.6780	529
5	2.626	0	Triplet-A	193 -> 196	0.6113	472
6	2.673	0	Triplet-A	192 -> 196	0.5723	464
7	2.916	0	Triplet-A	193 -> 197	0.5825	425
8	3.104	0	Triplet-A	195 -> 198	0.5939	399
9	3.179	0	Triplet-A	191 -> 196	0.4225	390
10	3.197	0	Triplet-A	189 -> 196	0.3760	388
11	3.413	0	Triplet-A	190 -> 196	0.4023	363
12	3.671	0	Triplet-A	195 -> 199	0.2845	338
13	3.694	0	Triplet-A	187 -> 196	0.4046	336
14	3.712	0	Triplet-A	187 -> 196	0.4191	334
15	3.773	0	Triplet-A	185 -> 196	0.4906	329
16	3.784	0	Triplet-A	184 -> 196	0.4597	328
17	3.959	0	Triplet-A	195 -> 199	0.3981	313
18	4.090	0	Triplet-A	186 -> 196	0.2991	303
19	4.104	0	Triplet-A	192 -> 197	0.3848	302
20	4.157	0	Triplet-A	188 -> 196	0.6018	298
21	4.188	0	Triplet-A	195 -> 199	0.3543	296
22	4.314	0	Triplet-A	194 -> 199	0.4199	287
23	4.386	0	Triplet-A	189 -> 197	0.4556	283
24	4.427	0	Triplet-A	187 -> 197	0.5394	280
25	4.493	0	Triplet-A	191 -> 197	0.4563	276
26	4.547	0	Triplet-A	191 -> 197	0.3650	273
27	4.576	0	Triplet-A	191 -> 198	0.4096	271

28	4.608	0	Triplet-A	191 -> 198	0.1996	269
29	4.658	0	Triplet-A	195 -> 200	0.3864	266
30	4.663	0	Triplet-A	185 -> 197	0.3282	266
31	4.748	0	Triplet-A	195 -> 200	0.3960	261
32	4.756	0	Triplet-A	193 -> 198	0.4874	261
33	4.816	0	Triplet-A	195 -> 201	0.2702	257
34	4.835	0	Triplet-A	183 -> 196	0.6679	256
35	4.853	0	Triplet-A	179 -> 196	0.2883	255
36	4.871	0	Triplet-A	194 -> 200	0.3746	255
37	4.886	0	Triplet-A	189 -> 198	0.2590	254
38	4.912	0	Triplet-A	189 -> 198	0.2735	252
39	4.950	0	Triplet-A	188 -> 197	0.3398	250
40	4.953	0	Triplet-A	195 -> 205	0.3061	250

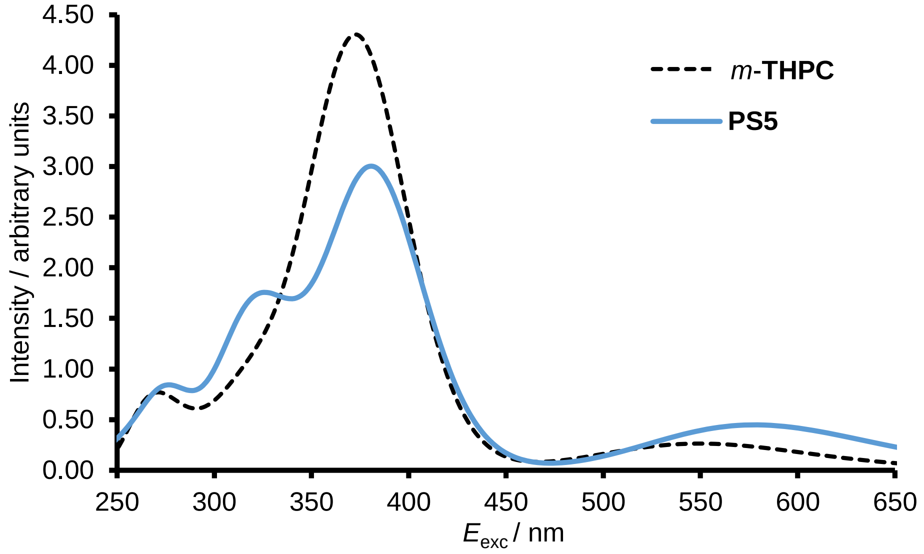

Figure S14. Comparison of the simulated UV/Vis spectra of *m*-**THPC** and investigated molecules **PS5**. The simulation is based on the BPE0/6-311+G(2d,p) calculations of excitation energies and oscillator strengths.

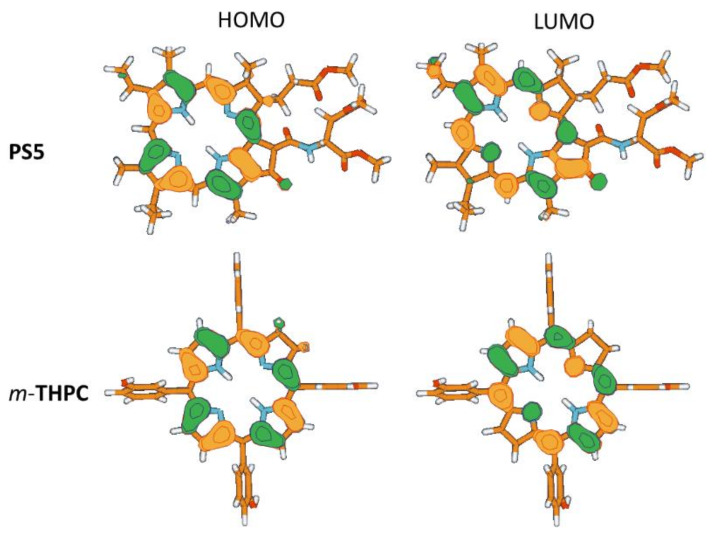

Figure S15. HOMO and LUMO Kohn-Sham orbitals of *m*-THPC and **PS5** at the PBE0/6-311+G(2d,p) level of theory.

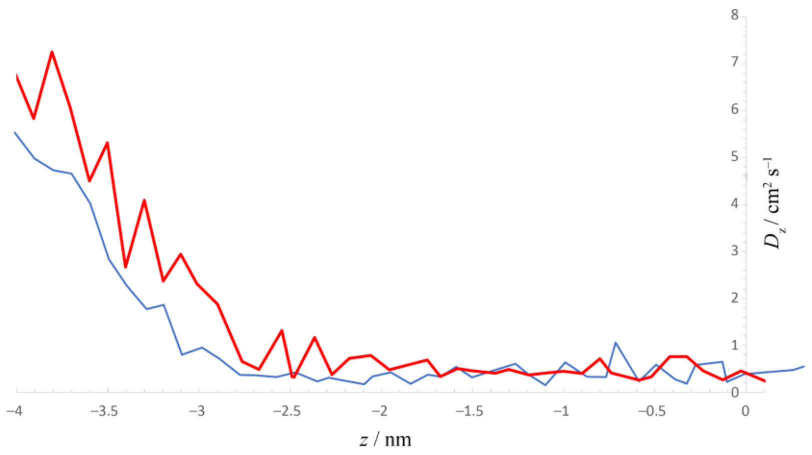

Figure S16. Position-specific diffusion coefficients for *m*-THPC (blue) and **PS5** (red) calculated via Hummer's positional autocorrelation extension of Wolf-Roux estimator.

Cartesian coordinates for optimized structures calculated at the B3LYP/6-311+G(2d,p) level of theory

m-**THPC**

C	4.358835	-3.552010	-1.455448
C	3.825105	-3.153621	-0.230476
C	4.364197	-3.673253	0.946767
C	5.424750	-4.575353	0.901651
C	5.958963	-4.968203	-0.322915
C	5.419973	-4.453224	-1.493667
C	2.686242	-2.180551	-0.167945
C	3.003684	-0.827875	-0.095069
N	2.125944	0.195422	-0.022925
C	2.799342	1.364419	0.029389
C	4.307543	1.167520	-0.019757
C	4.449613	-0.353384	-0.084313
C	2.237535	2.634501	0.113639
C	3.176485	3.802763	0.149606
C	3.663246	4.291991	1.361154
C	4.538720	5.375043	1.374526
C	4.937041	5.982947	0.192083
C	4.448787	5.499081	-1.019019
C	3.573804	4.415288	-1.039305
C	0.867800	2.945452	0.162790
N	-0.182929	2.071733	0.152212
C	-1.384292	2.752809	0.196490
C	-1.056016	4.142051	0.240258
C	0.306139	4.260144	0.223483
C	-2.655543	2.187380	0.176930
C	-3.808286	3.138409	0.241155
C	-4.136179	3.754663	1.449178
C	-5.206132	4.643408	1.520140
C	-5.957393	4.928725	0.383194
C	-5.628549	4.315082	-0.817892
C	-4.563051	3.422726	-0.896735
C	-2.941032	0.809628	0.098623
N	-2.019225	-0.194185	0.028038
C	-2.739638	-1.352056	-0.023764
C	-4.163020	-1.074366	0.011567
C	-4.288395	0.272031	0.094414
C	-2.204657	-2.652872	-0.111145
C	-0.851126	-2.971706	-0.161976
N	0.203030	-2.078608	-0.146078
C	1.397588	-2.741310	-0.183699
C	1.089444	-4.137779	-0.232270
C	-0.270848	-4.275626	-0.215481
C	-3.161272	-3.802089	-0.149272
C	-3.826211	-4.215523	1.004970
C	-4.708283	-5.291045	0.950453

C	-4.941912	-5.961970	-0.242273
C	-4.280565	-5.548274	-1.395487
C	-3.393540	-4.475560	-1.348926
H	1.827407	-4.921358	-0.269445
H	-0.835627	-5.192699	-0.229698
H	3.946648	-3.373145	1.902989
O	5.981815	-5.110994	2.031702
H	6.782366	-5.670695	-0.337668
H	4.787170	1.608088	0.855742
H	-1.781338	4.937752	0.271335
H	-4.949851	-1.810254	-0.021712
H	4.980042	-0.699119	-0.973011
H	0.885841	5.167584	0.245373
H	-5.197441	0.848828	0.146513
H	3.939354	-3.158492	-2.373055
H	-3.552888	3.533120	2.337124
H	-4.312659	2.945850	-1.835637
H	3.352135	3.825928	2.287882
H	3.190951	4.043878	-1.984950
H	-3.649584	-3.695103	1.937494
H	-2.880165	-4.154426	-2.249522
H	5.830653	-4.760944	-2.448071
O	-5.565471	5.266589	2.685367
H	-6.210367	4.536418	-1.704784
H	4.913323	5.752685	2.318608
O	4.862290	6.130547	-2.161166
H	-5.220163	-5.611828	1.849984
O	-4.541587	-6.234031	-2.551710
H	-6.784674	5.622735	0.458438
H	5.615354	6.826495	0.187592
H	-5.626882	-6.798238	-0.298624
H	0.086290	-1.076210	-0.098175
H	-0.112007	1.065052	0.104402
H	4.734668	1.672744	-0.888219
H	4.985843	-0.768009	0.771509
H	5.523132	-4.777821	2.811267
H	-4.984199	4.982104	3.399701
H	4.450448	5.718798	-2.929257
H	-4.038436	-5.850300	-3.278808

PS5

C	5.708033	1.344126	-0.576614
C	6.179675	2.666822	-0.927465
C	5.082803	3.505677	-0.974890
C	3.916453	2.717710	-0.671242
C	2.600776	3.167697	-0.632639
C	6.463976	0.198751	-0.428912
C	6.030529	-1.095049	-0.095320
C	6.930441	-2.240551	0.036380

C	6.145458	-3.301943	0.374063
C	4.779816	-2.787562	0.437513
C	1.438735	2.451180	-0.348874
C	0.100818	0.793009	0.220333
C	3.656464	-3.544187	0.757111
C	-0.214238	-0.504680	0.576266
C	0.707070	-1.559179	0.691063
C	0.135039	-2.800404	1.060444
C	1.143314	-3.756302	1.145290
C	2.349125	-3.067344	0.819072
H	2.466263	4.215417	-0.865514
H	7.525632	0.315402	-0.600483
H	3.797966	-4.593797	0.980473
N	4.749517	-1.448153	0.146112
N	1.389289	1.158683	-0.013510
C	-1.564304	-1.096282	0.972294
H	-1.894305	-0.687413	1.933429
C	-1.910819	2.221733	1.037314
H	-2.434739	1.313290	1.322917
H	-1.417172	2.580939	1.944690
C	-2.959041	3.230074	0.566368
H	-2.581594	4.256147	0.567796
H	-3.246020	3.026050	-0.470140
C	-1.289440	-2.616271	1.254405
O	-2.146691	-3.407014	1.593896
C	-2.723260	-0.900142	-0.005963
O	-2.597425	-0.361784	-1.098110
C	-4.234042	3.192686	1.379790
O	-4.438400	2.479001	2.331792
O	-5.146452	4.041479	0.872976
C	-5.147505	-1.078885	-0.256502
H	-5.030488	-1.351012	-1.306969
C	-6.446167	4.030325	1.505696
H	-6.344615	4.151003	2.583514
H	-6.983900	4.868942	1.070772
H	-6.959870	3.096094	1.279213
C	-0.253866	3.805924	-1.706562
H	0.437123	4.626383	-1.910561
H	-0.189698	3.096771	-2.534899
H	-1.264891	4.218343	-1.692360
C	1.011338	-5.201932	1.492259
H	0.000721	-5.417785	1.836812
H	1.215197	-5.835076	0.623367
H	1.715843	-5.489339	2.276943
C	8.410423	-2.202791	-0.176602
H	8.893445	-1.479137	0.486949
H	8.865603	-3.176000	0.009780
H	8.659267	-1.915523	-1.202853
C	6.547119	-4.729018	0.590236
H	7.598225	-4.773153	0.886679

H	5.983668	-5.149268	1.429127
C	6.333894	-5.605039	-0.655996
H	5.286737	-5.594879	-0.965883
H	6.928027	-5.233623	-1.493822
H	6.623780	-6.640942	-0.462735
C	5.063074	4.976184	-1.239860
H	4.921125	5.196130	-2.302947
H	4.256671	5.467888	-0.693729
H	6.004866	5.434373	-0.934318
C	8.073948	3.925382	-1.954177
H	7.438528	4.541808	-2.576642
H	9.141611	4.086614	-2.034750
C	7.585201	2.983911	-1.143011
H	8.294188	2.377234	-0.586658
N	2.028364	-1.738082	0.551535
H	2.744567	-1.065034	0.307462
N	4.344279	1.440260	-0.438409
H	3.738787	0.666762	-0.209579
C	0.065633	3.109910	-0.379127
H	0.024437	3.854819	0.425167
C	-0.874802	1.928328	-0.052762
H	-1.424346	1.635060	-0.951750
N	-3.919786	-1.317330	0.470218
H	-3.963756	-1.903594	1.295636
C	-5.518762	0.424256	-0.173949
H	-5.654517	0.716241	0.865270
H	-4.665809	0.967304	-0.585042
C	-6.243581	-1.934422	0.359616
O	-6.149591	-2.525473	1.405254
O	-7.346462	-1.911506	-0.401706
C	-6.742816	0.831249	-0.953195
O	-7.695407	1.410280	-0.487894
O	-6.631252	0.500426	-2.251431
C	-8.494722	-2.596256	0.140966
H	-9.282061	-2.468406	-0.596721
H	-8.268106	-3.651535	0.289340
H	-8.781159	-2.148910	1.092302
C	-7.765966	0.817784	-3.076307
H	-7.955417	1.891195	-3.067315
H	-7.503351	0.481013	-4.075781
H	-8.651216	0.295059	-2.713210

References

1. Sharp, D.; Forsythe, S.; Davis, J., Electrochemical Monitoring of Singlet Oxygen Production. *Electroanalysis* **2009,** *21* (21), 2293-2296.
2. Tang, W.; Xu, H.; Kopelman, R.; Philbert, M. A., Photodynamic characterization and in vitro application of methylene blue-containing nanoparticle platforms. *Photochem. Photobiol.* **2005,** *81* (2), 242-249.
